# Modelling ischaemic AKI in human kidney organoids reveals injury-associated epithelial states and macrophage-epithelial crosstalk

**DOI:** 10.1186/s13073-026-01712-z

**Published:** 2026-08-19

**Authors:** Ana B. Nunez-Nescolarde, Yang Liao, Laura Perlaza-Jiménez, Mehran Piran, Zhengqi Cheng, Chris K. Barlow, Joel R. Steele, Deanna Deveson, Julie L. M. Moreau, Han-Chung Lee, Jinhua Li, Ralf B. Schittenhelm, Christine A. Wells, Wei Shi, David J. Nikolic-Paterson, Alexander N. Combes

**Affiliations:** 1 Department of Anatomy and Developmental Biology, Development and Stem Cells Program, Monash Biomedicine Discovery Institute, Monash University, Clayton, Victoria 3800 Australia; 2 Department of Biochemistry and Molecular Biology, Cancer Program, Monash Biomedicine Discovery Institute, Monash University, Clayton, Victoria 3800 Australia; 3 Monash Bioinformatics Platform, Monash University, Clayton, Victoria 3800 Australia; 4 Department of Anatomy and Physiology, Faculty of Medicine, Dentistry and Health Sciences, The University of Melbourne, Melbourne, Victoria 3010 Australia; 5 Monash Proteomics and Metabolomics Facility, Monash University, Clayton, Victoria 3800 Australia; 6 Department of Nephrology, Guangdong Provincial People’s Hospital, Guangdong Academy of Medical Sciences, Guangzhou, 510180 China; 7 Department of Nephrology, Monash Health and Monash University Centre for Inflammatory Diseases, Monash Medical Centre, Clayton, Victoria 3168 Australia

**Keywords:** Acute kidney injury, Kidney organoids, Ischemic acute kidney injury, Injury-associated cell states, Macrophages, Epithelial repair, Hypoxia, Metabolic reprogramming, Single-cell transcriptomics, Spatial transcriptomics

## Abstract

**Background:**

Acute kidney injury (AKI) is a common clinical syndrome associated with high morbidity and progression to chronic kidney disease. Ischaemia is a leading cause of AKI, driving cellular stress, metabolic reprogramming, and injury-associated epithelial states. Scalable human models that enable controlled investigation of ischaemic injury, repair, and therapeutic targets in AKI remain limited. We therefore assessed the extent to which induced pluripotent stem cell (iPSC)-derived human kidney organoids recapitulate key features of ischaemic AKI.

**Methods:**

Kidney organoids were subjected to hypoxic injury (1% O₂, 48 h) followed by normoxic recovery. Transcriptomic, proteomic, metabolomic, single-cell, and spatial profiling were performed across acute injury and recovery phases. iPSC-derived macrophages were integrated into organoids and analysed following hypoxic injury.

**Results:**

Hypoxia induced acute stress responses, including hypoxia-inducible factor activation, glycolytic reprogramming, cell cycle arrest, and induction of injury markers. Following recovery, organoids exhibited sustained inflammatory signalling and persistent metabolic dysregulation. Single-cell analysis revealed loss of cell type-specific markers and key functional genes across nephron segments. After return to normoxia, podocyte and distal tubule markers were largely restored, whereas proximal tubule markers showed only partial recovery. Injury-associated and inflammatory programs persisted across all nephron cell types, including upregulation of GDF15, MMP7, SPP1, CXCL2, and ICAM1, with enrichment of complement, TNF-NFκB, and lipid-associated inflammatory pathways. Injured proximal tubules were enriched for adaptive/maladaptive repair signatures derived from human kidney biopsies and displayed heterogeneous recovery. While some cells restored canonical identity, others retained dedifferentiated injury-associated states, including focal expression of CDKN1A and VCAM1. Integrated macrophages transitioned from homeostatic, resident-like profiles to activated phenotypes following injury, exhibiting spatially localised interactions with injured tubules and increased expression of cytokines, chemokines, and matrix-remodelling factors.

**Conclusions:**

Human kidney organoids recapitulate key epithelial features of hypoxic injury, including segment-specific vulnerability, persistent inflammatory signalling, and heterogeneous recovery, with integrated macrophages adopting activated inflammatory states following injury. While constrained by developmental immaturity, this system provides a tractable human platform to investigate injury-associated epithelial states and macrophage–epithelial crosstalk in AKI.

**Supplementary Information:**

The online version contains supplementary material available at 10.1186/s13073-026-01712-z.

## Background

AKI affects over 13 million people annually and is associated with substantial morbidity, healthcare costs, and more than 1.7 million deaths worldwide [1, 2]. Ischaemia is a leading cause of AKI, arising in settings including cardiovascular events, urinary tract obstruction, sepsis, kidney transplantation, and major surgery [3–5]. Although kidney function can recover following mild to moderate injury, AKI is strongly associated with an increased risk of long-term kidney dysfunction and progression to chronic kidney disease (CKD) [6]. Current management remains largely supportive, underscoring a major unmet need for therapies that target the underlying mechanisms of injury and repair.

Ischaemic injury disrupts oxygen and nutrient delivery to the kidney, triggering epithelial stress responses, inflammation, and metabolic reprogramming, with the proximal tubule being particularly susceptible [7]. Injured tubules adopt dynamic injury-associated states characterised by dedifferentiation, proliferation, and activation of stress and inflammatory pathways [8–11]. These responses can support adaptive repair, culminating in re-establishment of tubular identity and function. However, persistent injury-associated epithelial states can also emerge, marked by sustained inflammatory signalling, profibrotic programs, and metabolic dysfunction, which are linked to disease progression [12–16]. Tissue-resident and recruited macrophages play central roles in shaping these outcomes, responding to injury and influencing epithelial repair or inflammatory amplification depending on the context [17, 18].

Much of our mechanistic understanding of AKI has been derived from experimental models, particularly in rodents, which have been instrumental in defining injury pathways and repair processes. Complementing these approaches, recent advances in single-cell and spatial transcriptomics, including large-scale efforts such as the Kidney Precision Medicine Project (KPMP), have enabled characterisation of injury-associated cell states in human kidney biopsies [19–26]. These studies have provided important insights into epithelial heterogeneity and injury-associated transcriptional programs in human disease. However, they do not readily capture the temporal dynamics of injury, recovery, or cell-cell interactions.

Human kidney organoids provide a complementary platform for modelling kidney injury in a human context. Recent studies have demonstrated their utility in modelling nephrotoxicity, inflammatory stressors, and other aspects of kidney disease [27–34]. While organoids do not fully recapitulate mature cell states, they enable interrogation of conserved injury-associated responses under controlled conditions with potential for high-throughput and combinatorial perturbation studies that are not feasible in animal models or clinical cohorts. Here, we investigate the extent to which human iPSC-derived kidney organoids recapitulate epithelial and metabolic responses to hypoxic injury at tissue and cellular resolution. We further incorporate iPSC-derived macrophages to interrogate macrophage-epithelial interactions during injury and recovery.

## Methods

### Study design and experimental cohorts

This study used previously established human induced pluripotent stem cell (iPSC) lines and did not involve recruitment of new donors. Three established human iPSC lines (522.3, 808.5 and D4C4), derived from unrelated healthy donors as described below, were used to establish the hypoxic injury model. Following confirmation of reproducible responses across all three lines, subsequent multi-omic analyses were performed using the 522.3 iPSC line. Unless otherwise stated, all organoids within each omics experiment were generated from a single differentiation to minimise biological and technical variability, with the spatial transcriptomic study incorporating two independent differentiations to increase biological replication. Kidney organoids were generated as described below and subjected to hypoxic injury (1% O₂, 48 h) from day 18 to day 20 (d20h), with stage-matched normoxic controls (d20n). Recovery was assessed following five days of normoxia (d25h/n), with corresponding day 25 normoxic controls (d25n). Human iPSC-derived macrophages generated from the PB001.1 iPSC line were incorporated into kidney organoids at day 7 (5% macrophages) and assessed at day 20 and day 25 to investigate macrophage-epithelial interactions following injury.

Bulk RNA sequencing was performed using three biological replicates per condition (two pooled organoids per replicate). Single-cell RNA sequencing was performed using three biological replicates per condition (three pooled organoids per replicate). Proteomic profiling was performed on four individual organoids per condition, while untargeted metabolomic profiling was performed on eight organoids per condition. Spatial transcriptomic analysis included 2–6 organoids per condition generated across two independent differentiations. Human iPSC-derived macrophages were analysed by flow cytometry prior to integration and by bulk RNAseq (3 replicates per group, each containing two pooled organoids) and spatial transcriptomics (4 organoids per group) following integration into injured and control organoids.

Together, this experimental design enabled integrated transcriptomic, proteomic, metabolomic, single-cell and spatial analyses of acute hypoxic injury, recovery and macrophage-epithelial interactions in human kidney organoids. Detailed methodologies, sample preparation, analytical workflows and statistical analyses for each experimental modality are provided in the corresponding sections below.

### iPSC culture and organoid generation

The following human iPSC lines were used: 522.3 (522, female donor; derived from GM04522 fibroblasts), 808.5 (male donor; derived from GM21808 foreskin fibroblasts) - both derived by the NIGMS Human Genetic Cell Repository. D4C4 (female donor; derived from gingival tissue). Cells were maintained in Essential 8 (E8) media with Essential 8 supplement (Thermo Fisher Scientific) supplemented with 1% Penicillin/Streptomycin (Pen/Strep) (Thermo Fisher Scientific) in 6-well plates coated with Matrigel (Corning, cat no. 354277). Cells were passaged at 70–80% confluency at 1:3 ratio using 0.5 mM EDTA (Thermo Fisher Scientific). Human iPSC were directed to differentiate into kidney organoids using the Takasato protocol [32, 35, 36], with minor modifications (Additional file 1) and predefined morphology-based quality control criteria (Additional file 1: Fig. S1). Details on organoid embedding, sectioning, immunofluorescence, antibodies, staining reagents and image analysis workflows are provided in Additional file 1. Dagmar Wilhelm generously provided a custom sheep anti-SOX9 antibody used in these studies; details of its generation and validation are described in [37].

### iPSC-derived macrophage differentiation

Macrophages were differentiated from MCRIi0001-A (PB001.1) human iPSCs based on established protocols [38–41]. In short, the PB001.1 line was maintained in E8 medium with Essential 8 supplement (Thermo Fisher Scientific) on growth factor-reduced Matrigel (Corning, cat. no. 356234). Embryoid bodies (EBs) were formed as per Ng et al., [39], and differentiated towards the monocyte/macrophage lineage as per Joshi et al. [38], with the following modifications: E8 was used as the base media, EBs were differentiated in suspension culture and myeloid progenitors were collected from day 11, and detail on cytokines and factors used to differentiate iMacs are provided in Additional file 1. Myeloid progenitors were filtered through a 70 μm mesh and cultured in RPMI-1640 containing L-glutamine (Life Technologies), 10% fetal bovine serum, and 100 ng/mL CSF1 (R&D Systems, cat. no. 216-MC-500) for four days to promote macrophage differentiation. Successful differentiation was defined by a CD45+/CD14+/CD68 + phenotype (> 95% purity), assessed via flow cytometry (BioLegend, cat. nos. CD14 301830, CD45 368512, CD68 333816). iMacs were combined with dissociated cells from 522 iPSC kidney monolayer differentiations at day 7 (5% iMacs, 95% kidney differentiation) and centrifuged in 1.5 mL Eppendorf tubes to generate iMac-containing organoids, which were subsequently cultured as per controls.

### Hypoxic injury

Hypoxic injury was modelled by culturing d18 organoids in 1% O_2_ for 48 h, repair was evaluated in d25 injured organoids after a 5-day recovery in 19% O_2_. Comparisons were made to stage-matched control organoids cultured in 19% O_2_. Four organoids were cultured per transwell across all experiments to minimize potential variability in the diffusion and metabolism of nutrients and oxygen.

### Bulk and single cell RNAseq

For bulk RNAseq, mRNA was extracted from 3 replicate samples, each composed of a pool of two organoids for each experimental group. All organoids in the experiment were generated from a single differentiation of the 522 iPSC line. RNA was sequenced using a 3’-Multiplexed method [42]. Data was normalized using the counts per million (CPM) method, differentially expressed (DE) genes were defined using voom and limma R packages [43, 44] in Degust – an interactive RNAseq analysis platform available at https://degust.erc.monash.edu [45] (BH adjusted *p*-value < 0.05 & absolute LogFC > 0.5). Enrichment analysis was performed with DAVID [46] and Gene Set Enrichment Analysis [47]. Cell type enrichment was assessed by hypergeometric over-representation analysis comparing differentially expressed genes with a false discovery rate (FDR) < 0.05, against kidney cell type gene signatures: Podocyte (Pod), proximal tubule (PT), Loop of Henle (LoH), distal tubule (DT), Stroma, Endothelial; ~136 genes each. Signatures were derived from a developing human kidney single cell RNA sequencing (scRNAseq) reference [48], and tested using Bonferroni correction. Further details on data processing, quality control and analysis are provided in Additional file 1. Bulk RNAseq data are available from NCBI under accession GSE236379 (https://www.ncbi.nlm.nih.gov/geo/query/acc.cgi?acc=GSE236379). Bulk RNAseq data from iPSC-derived macrophage co-culture experiments are available under accession GSE278562 (https://www.ncbi.nlm.nih.gov/geo/query/acc.cgi?acc=GSE278562).

For scRNAseq, 3 iPSC-derived kidney organoids were pooled per replicate, with a total of 3 replicates per condition for d20n, d20h, d25h/n, d25n. All organoids were derived from a single differentiation of the 522 iPSC line. Replicates were individually labelled with CellPlex reagents (10x Genomics) and processed on the day of collection. Gene expression and feature barcode libraries were prepared using Chromium Single Cell 5’ V3.1 chemistry (10x) and sequenced on MGITech MGISEQ2000RS hardware using MGIEasy V3 chemistry for 100 bp paired-end sequencing. Quality control and analysis was performed using the R package Seurat (v. 4.3.0) [49] and related tools. scRNAseq data are available under accession GSE242425 (https://www.ncbi.nlm.nih.gov/geo/query/acc.cgi?acc=GSE242425). scRNAseq quality control, analysis workflows and associated code are available through GitHub (https://github.com/MonashBioinformaticsPlatform/sc-hyp-org) and archived on Zenodo (https://zenodo.org/records/20840256). An interactive web application for exploration of the single-cell dataset, including cell cluster visualisation, gene expression and marker gene analysis, is available at https://bioinformatics.erc.monash.edu/apps/hypoxia-kidney-organoid/. PT cells were redefined using module scoring based on 20 canonical PT marker genes. Cells with scores exceeding mean + 1 SD and originating from PT, DT, or early nephron clusters were classified as PT, and reclassification was supported using an independent 28-gene PT marker set. Differential expression between d25n and d25h/n redefined PT cells was performed using a pseudobulk DESeq2 approach, aggregating raw counts at the level of biological replicates. Gene set scores were calculated using Seurat’s AddModuleScore function against KPMP-derived PT and adaptive PT signatures (Lake et al., Nature 2023, Supplementary Table 11) [19]. Subpopulation analysis was performed by reclustering d25h/n redefined PT cells at resolution 0.8. An independent PT reclassification using UCell-based module scoring and more stringent marker thresholds was performed as a sensitivity analysis and produced consistent findings. Full details of both PT reclassification approaches, including marker sets, thresholds and validation analyses, are provided in Additional file 1.

### Metabolomic and proteomic analysis

Liquid chromatography-mass spectrometry (LC-MS) proteomic and metabolomic profiling were performed on matched organoid samples, all derived from a single differentiation of the 522 iPSC line. Proteomic analysis was conducted on *n* = 4 organoids/group, using an Orbitrap Fusion Tribrid mass spectrometer (Thermo Fisher Scientific) coupled to a Dionex Ultimate 3000 RSLCnano system. Raw data were analyzed with the Fragpipe software suite [50], and LFQ-Analyst was used for downstream statistical analysis [51]. Significant changes were defined in pairwise comparisons using adjusted p $\:\le\:$ 0.05 (Benjamini-Hochberg method) and log2 fold change $\:\ge\:$ 0.5 or $\:\le\:$ -0.5 thresholds. 8 organoids per group were profiled for untargeted metabolomic analysis to account for higher variability in this method, using a Dionex RSLC3000 UHPLC coupled to a Q-Exactive Plus MS (Thermo Fisher Scientific). Sample extraction and analysis details provided in Additional file 1. Proteomics data are available via the PRIDE database [52] (ProteomeXchange Consortium) under accession PXD042882 (https://www.ebi.ac.uk/pride/archive/projects/PXD042882), metabolomics data are available through Store.Monash, accession 16,985 (https://store.erc.monash.edu.au/experiment/view/16985/).

### Representative Xenium imaging dataset

To illustrate the cellular composition of day 20 human kidney organoids under normoxic conditions, a representative spatial transcriptomic dataset was generated using the Xenium Human Multi-Tissue Atlassing Panel (10x Genomics). Two day 20 organoids derived from the 522 iPSC line were processed using the standard Xenium workflow and visualised in Xenium Explorer. The processed Xenium output directory has been deposited in Zenodo to enable interactive visualisation of tissue morphology, cell segmentation and spatial gene expression 10.5281/zenodo.20889605. This dataset was not used for the pseudobulk differential expression analyses presented in this study.

### Spatial transcriptomic analysis of hypoxic injury and iMac-integrated organoids

Kidney organoids +/- integrated iMacs were generated and subjected to hypoxic injury as described above. iMacs were derived from one differentiation of the PB001.1 iPSC line. Experimental groups comprised 2–6 kidney organoids per condition, derived from two independent differentiations of the 522 iPSC line. Spatial slide identifiers that follow (32459, 32419) refer to Xenium capture slide IDs used for profiling and are not batch or differentiation identifiers. Most conditions were derived from one differentiation and profiled on spatial slide 32,459, including d20n (32459.d20n, *n* = 2), d20h (32459.d20h, *n* = 3), d25n (32459.d25n, *n* = 2), and d25h/n (32459.d25h/n, *n* = 3). Macrophage co-culture conditions were derived in the same differentiation but profiled on a separate slide (32419), including d25n+iMacs (32419.d25n_5pct_imacs, *n* = 4) and d25h/n+iMacs (32419.d25h_5pct_imacs, *n* = 4). Additional d25 samples were included from an independent differentiation to support reproducibility and boost sample number at this critical stage, comprising d25n (32419.d25n, *n* = 4) and d25h/n (32419.d25h/n, *n* = 3). 5 μm paraffin sections of samples were profiled with the 380-gene Xenium Human Immuno-Oncology panel, using the Xenium Cell Segmentation kit (10X Genomics), as per the manufacturer’s protocols. These data are available through NCBI under accession GSE307588 (https://www.ncbi.nlm.nih.gov/geo/query/acc.cgi?acc=GSE307588). Data were imported into R, loaded into a Seurat object, and normalized with SCTransform (v. 4.3.0) [37]. Principal Component Analysis was performed on the SCT-normalized data, and the top 100 components were used for uniform manifold approximation and projection (UMAP) visualization. Cell type identities were assigned to Xenium clusters by integrating our organoid scRNAseq reference using RCTD [53] and Seurat’s anchor-based transfer, with results cross-validated and refined by manual assessment of clusters and marker gene expression on tissue images. Pseudobulk differential expression (DE) analysis was performed on log₂-transformed, quantile-normalized, and precision-weighted data with voom (limma) [43, 44]. Linear models with empirical Bayes moderation [54] were used to identify DE genes at FDR ≤ 0.05 for comparisons at the level of sample and cell lineage. Cluster-based DE was performed on the single cell level with an FDR cutoff for DE ≤ 0.2. Targeted DE analysis was performed on twenty-four manually defined regions of interest (ROIs) containing injured nephrons +/-iMacs in the d25h/n_iMacs organoid group, with cells selected by centroid location. The same pseudo-bulk limma-voom pipeline (FDR ≤ 0.05) was used to compare all cells, non-iMac cells, and nephron cells only between +/-iMac ROIs. Further details are provided in Additional file 1.

### Statistical analysis

Details on sample number, statistical analyses and cutoff thresholds for each experiment are included in Results, Figure legends, Methods and Additional file 1. All data points were included in the statistical analysis and displayed in related graphs where possible.

## Results

### Hypoxia induces markers of injury and inflammation in human kidney organoids

**Fig. 1:**
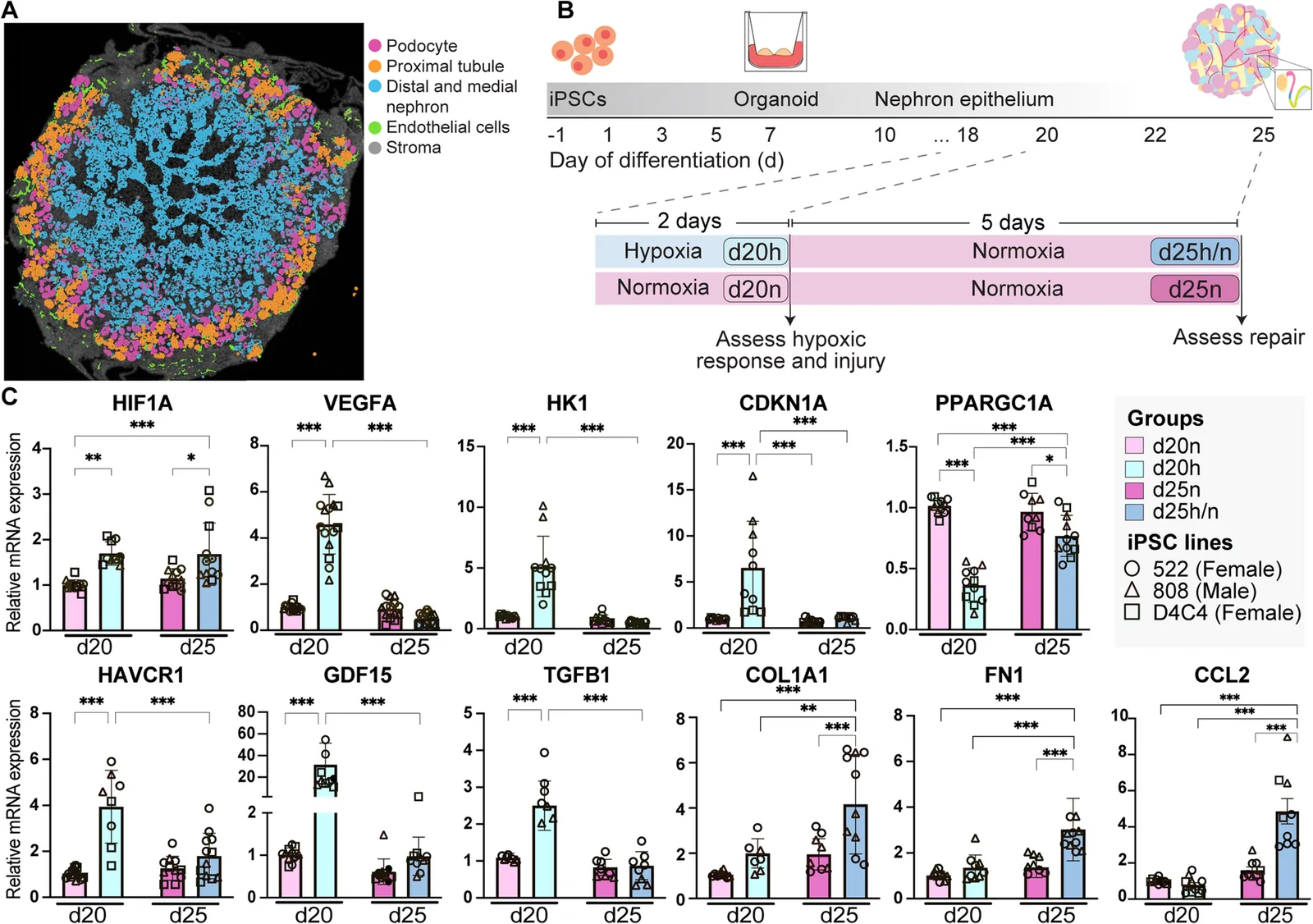
Evaluating hypoxic injury in kidney organoids from three iPSC lines** A** Distribution of podocyte (pink), proximal tubule (orange), distal/medial nephron (blue), endothelial cells (green), and stroma (grey) in Xenium spatial data from a representative day 20 kidney organoid **B** Overview of experiment, illustrating differentiation and organoid generation from d-1 to d18, hypoxic exposure (d18-20), normoxic recovery (d20-d25), and sample collection points at d20 and d25 **C** qPCR analysis of hypoxic response genes (*HIF1A*,* VEGFA*,* HK1*), markers of cell cycle arrest (*CDKN1A*), mitochondrial function (*PPARGC1A*), kidney injury (*HAVCR1/KIM1*,* GDF15*,* TGFB1*), extracellular matrix (*COL1A1*,* FN1*) and inflammation (*CCL2*). Data shown as mean with SD; *n* = 7–10 from three independent iPSC lines. Y axis shows mRNA expression normalised to *ACTB* within each sample, relative to d20n mean. Analyses were performed by one-way ANOVA with Tukey’s multiple comparison test: *p < 0.05, **p < 0.01, ***p < 0.001

Kidney organoids were generated from human iPSC lines using the Takasato protocol [32, 35, 55] (Fig. 1A). To define a hypoxic injury paradigm, we evaluated exposure durations and found that 24 h hypoxia (1% O₂) induced transient transcriptional responses that resolved upon return to normoxia, whereas 48 h produced sustained changes (Additional file 1: Fig. S1). Guided by mouse AKI models, where divergence between resolving or persistent injury states becomes apparent over ~ 3–7 days, and by known confounding effects of extended culture of the Takasato organoid system beyond day 26 [36, 56], we selected a 48 h exposure followed by a 5-day recovery period to assess resolution versus persistence of injury responses.

Based on this framework, organoids were exposed to hypoxia from day 18 to day 20 and analysed following recovery at day 25 (Fig. 1B). Although nephron maturation continues after d18, organoids at this stage expressed markers of established stromal, podocyte, proximal and distal nephron cell types (Additional file 1: Fig. S1). This is consistent with prior temporal profiling showing major gains in nephron marker expression occur before day 18, with relatively stable expression between day 18 and 25 [57]. This design allowed interrogation of hypoxia-induced responses in established epithelial populations within a developmentally immature epithelial context.

Organoids were collected at defined timepoints to capture acute and persistent phases of the response (Fig. 1B). Acute effects were assessed immediately following hypoxic exposure at day 20, while later responses were evaluated after return to normoxia at day 25. Experimental groups are defined as: day 20 hypoxia (d20h); day 20 normoxic control (d20n); day 25 following hypoxia and normoxic recovery (d25h/n; ‘injured’); and day 25 normoxic control (d25n). Comparisons were performed against stage-matched normoxic controls to account for temporal changes in organoid maturation and composition.

To determine whether kidney organoids mount a robust and reproducible response to hypoxia, initial experiments establishing the model were performed across three independent iPSC lines. Ischaemic AKI is characterized by a HIF1A-mediated hypoxic response [58, 59], tubular injury and cell cycle arrest [60]. A robust hypoxic response was evident in organoids exposed to 1% O_2_ with stabilization of HIF1A protein (Additional file 1: Fig. S1D) and increased expression of HIF1A target genes *VEGFA* and the glycolytic enzyme hexokinase 1 (*HK1*), as measured by quantitative PCR (qPCR) across multiple differentiations from three iPSC lines (Fig. 1C). The mitochondrial biogenesis regulator *PPARCG1A* was significantly reduced in d20h kidney organoids, mirroring similar findings in human AKI [61]. Injury-associated epithelial responses were evident in d20h organoids, with upregulation of cell cycle arrest marker *CDKN1A* (also known as p21), injury markers *HAVCR1 (KIM1)* and *GDF15*, accompanied by increased *TGFB1* which is associated with reparative and fibrotic signalling (Fig. 1C) [60, 62, 63].

Following return to normoxia, d25h/n organoids exhibited sustained induction of inflammatory and matrix-associated genes, including *CCL2*,* COL1A1*, and *FN1*, consistent with persistent injury-associated states described in human AKI. In contrast, shorter hypoxic exposure (24 h) induced transient injury responses (e.g. *VEGFA*,* GDF15*) without upregulation of inflammatory or matrix-associated genes at d25 (Additional file 1: Fig. S1D), supporting a duration-dependent injury response in this model. Having established a conserved response across iPSC lines, subsequent experiments were performed in a single line to minimise technical variability and experimental complexity.

### Transcriptional profiling identifies injury-associated and inflammatory states in organoids following hypoxic injury

**Fig. 2:**
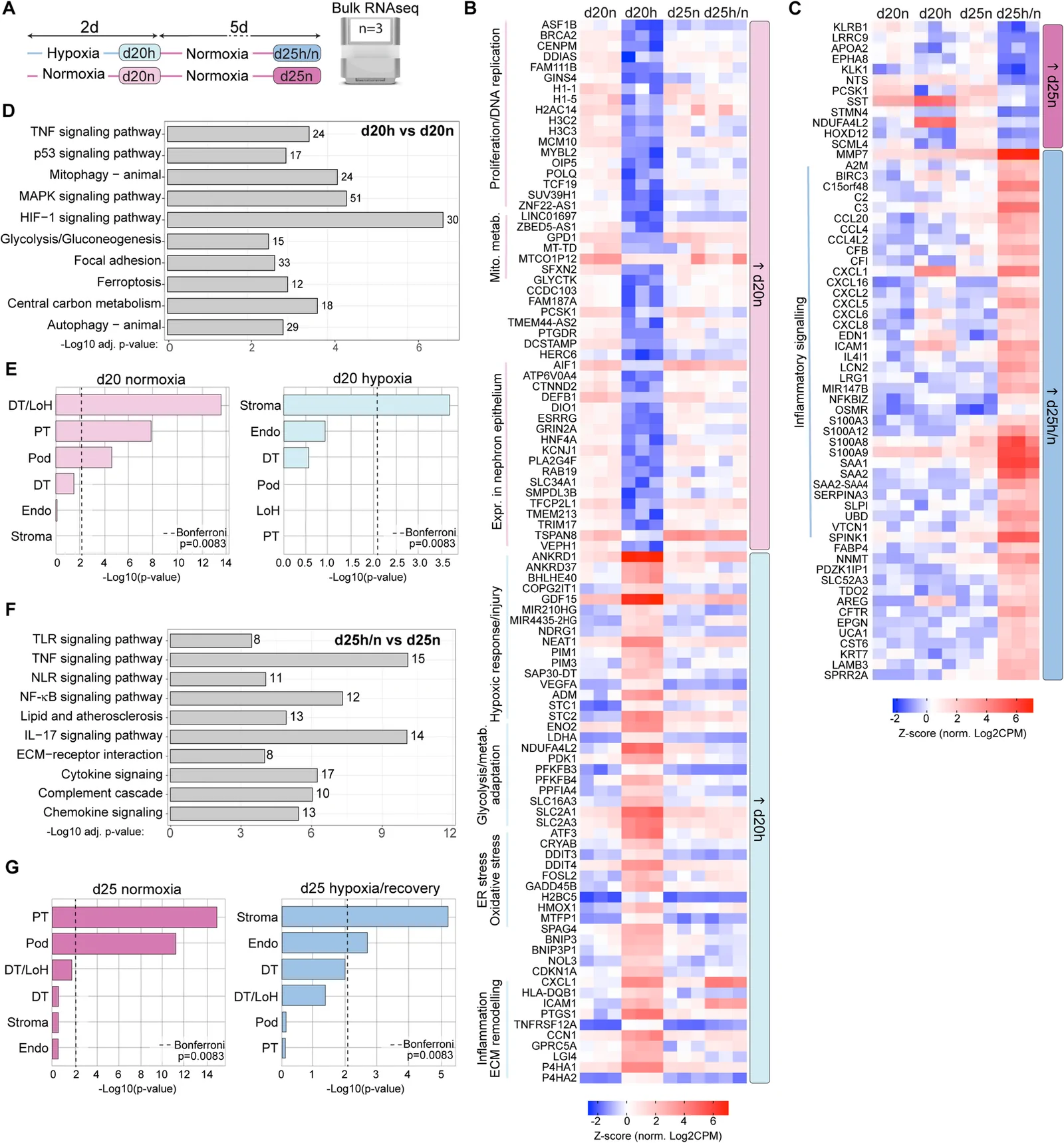
Transcriptional analysis of hypoxic injury and repair in kidney organoids **A** Overview of bulk RNAseq experiment. *n* = 3 biological replicates per experimental group, each comprising two pooled organoids; all organoids were derived from a single differentiation **B** Top 50 up- and downregulated genes from differential expression analysis between d20n and d20h organoids (absolute LogFC > 2, ranked by FDR), grouped by broad biological function or expression in scRNAseq reference data **C** Top up- and downregulated genes between d25n and d25h/n organoids (absolute LogFC > 2, ranked by FDR), grouped by broad biological function for plotting **D** KEGG enrichment analysis between d20h and d20n. Number of DE genes shown for each term **E** Enrichment analysis of human kidney cell type signatures in d20 DE genes (absolute LogFC > 0.58, FDR < 0.05). Significance threshold (Bonferroni *p* < 0.0083) indicated by dotted line. DT/LoH, distal tubule/Loop of Henle; PT, proximal tubule; DT, Distal tubule; Pod, Podocyte; Endo, endothelial **F** Upregulated KEGG pathways in d25h/n compared to d25n organoids **G** Enriched kidney cell type signatures in d25h/n and d25n organoids. Abbreviations and dotted line as per E

To gain a deeper understanding of responses to prolonged hypoxic exposure, we performed bulk RNA-sequencing (RNAseq), assessing differential expression between injured and control groups at d20 and d25 (Fig. 2A-C, Additional file 2). Top upregulated genes in d20n control organoids were associated with cell proliferation, mitochondrial metabolism, or predominantly expressed in nephron cell types in human kidney scRNAseq reference data [48] (Fig. 2B). In contrast, top upregulated genes in the d20 hypoxic group were linked to the hypoxic response, glycolysis, endoplasmic reticulum and oxidative stress (*HMOX1*), cell cycle arrest (*CDKN1A*), cell death (*JUN*,* FOS*,* BNIP3*) and inflammation (Fig. 2B), with these signatures reinforced by KEGG pathway analysis (Fig. 2D). Enrichment of mitophagy, glycolysis and central carbon metabolism pathways suggest mitochondrial dysfunction and a shift towards oxygen-independent energy production (Fig. 2D). Analysis of kidney cell type signatures among differentially expressed genes showed enrichment of distal tubule/Loop of Henle, proximal tubule, and podocyte markers in control organoids. In contrast, stromal signatures were increased following hypoxic exposure, suggesting altered epithelial identity and stromal expansion (Fig. 2E).

Comparison of injured d25h/n organoids to stage-matched d25n controls revealed significant upregulation of genes associated with inflammatory signalling and injury-associated epithelial states including chemokines (*CCL2/MCP-1*,* CCL4/20*,* CXCL1/2/5/6/8/16*), transcriptional regulators (*IRF1*, *STAT1/3*, *IL32*, *NFKBIA/Z*), and markers of tubular injury (*LCN2/NGAL*, *ICAM1*,* VCAM1*,* SPP1*,* LGALS3*, *S100A8/9*) (Fig. 2C, Additional file 2). Genes associated with extracellular matrix remodelling including *MMP7/9*,* PCOLCE*, and *INHBA* were also increased. KEGG pathway analysis identified enrichment of inflammatory programs including the TLR, TNF and NF-κB signalling, interleukin and chemokine signalling, complement activation and extracellular matrix interactions (Fig. 2F). Proximal tubule and podocyte cell type signatures were enriched in d25n control organoids, consistent with nephron maturation over time, while increased stromal and endothelial signatures in injured d25h/n organoids suggest progressive stromal expansion and tissue remodelling following injury (Fig. 2G).

### Proteomic profiling affirms injury-associated and inflammatory signatures

**Fig. 3:**
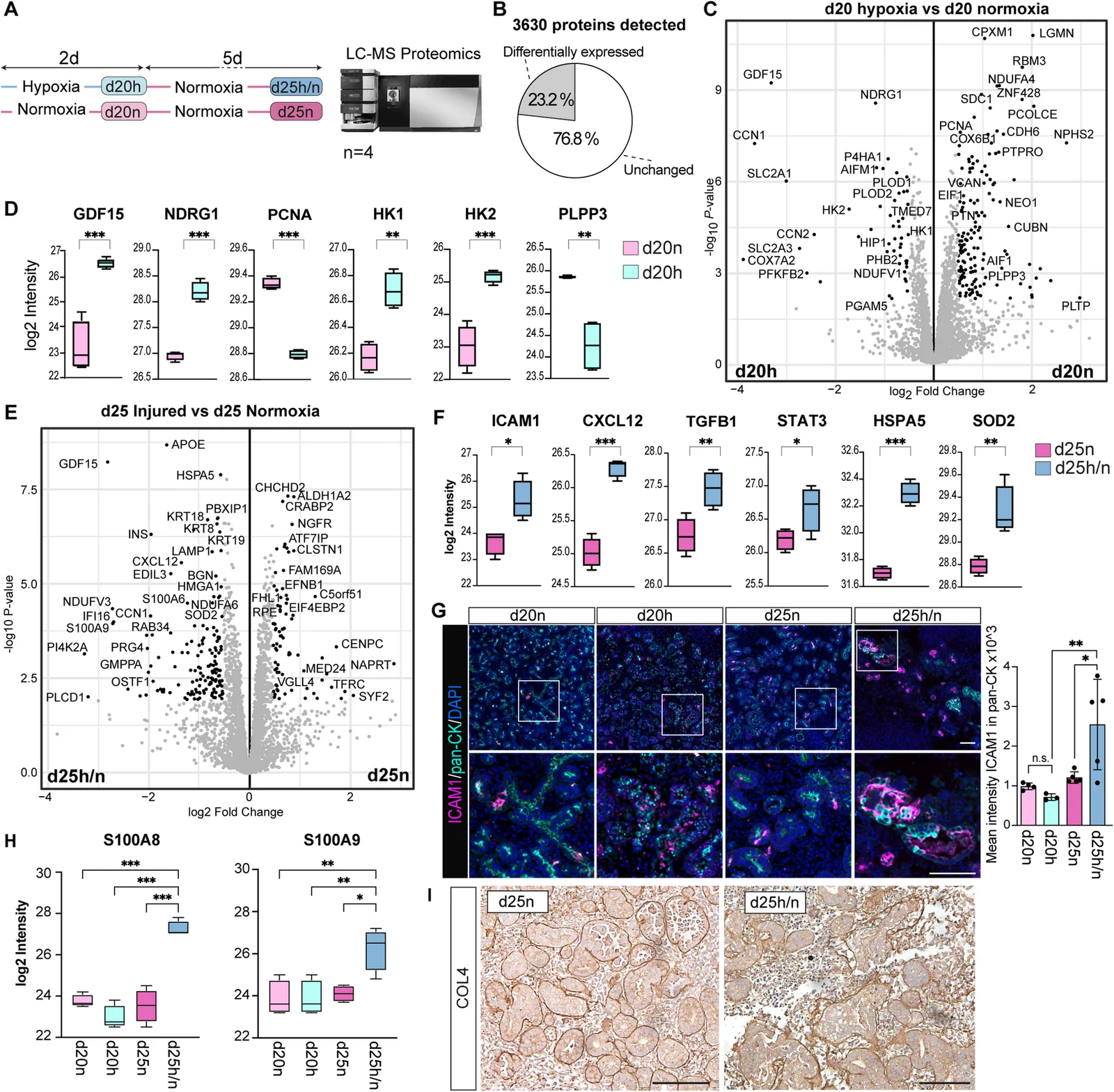
Proteomic analysis of hypoxic injury and repair **A** Overview of MS-based proteomics experiment (*n* = 4 organoids per group, all derived from a single differentiation) **B** Pie chart of differentially regulated proteins across all conditions **C** Volcano plot of significantly changed proteins at d20 **D** Intensity plots of proteins related to injury (GDF15), hypoxic response (NDRG1), cell cycle (PCNA) and metabolism (HK1, HK2, PLPP3) at d20 **E** Volcano plot of significantly changed proteins at d25 **F** Intensity plots of key proteins related to inflammatory and profibrotic processes (ICAM1, CXCL12, TGFB1, STAT3) and oxidative stress (HSPA5, SOD2) at d25 **G** Immunofluorescence for pan-cytokeratin (pan-CK, tubules), ICAM1 (inflammatory marker) and DAPI. Bottom panels show magnified regions indicated by white box in upper panels. Scale bars, 100 μm for all images (shown in right hand panel). Mean ICAM1 fluorescence intensity in tubules was quantified; data points mean values from *n* = 4 organoids per group, all from a single differentiation, which was distinct from the differentiation used for proteomic analysis. Differences between groups were assessed using two-tailed Welch’s t-tests **H** Markers of maladaptive repair S100A8 and S100A9 are upregulated in d25h/n organoids **I** Representative image of collagen type 4 (COL 4) staining in d25h/n and d25n organoids from an experiment containing *n* = 4 organoids per group, derived from a single differentiation. Scale bar, 200 μm. Asterisks and annotations for bar charts: n.s. non-significant, *p < 0.05, **p < 0.01, ***p < 0.001

To examine injury responses at the protein level, we performed untargeted LC-MS-based proteomics on 4 replicate samples from each experimental group (Fig. 3A and Additional file 1: Fig. S2). A total of 844 proteins were significantly altered across conditions from 3630 proteins quantified (Fig. 3B and Additional file 3). GDF15 was significantly increased in d20h organoids, consistent with its induction during ischaemic injury and tubular damage in animal models and human kidney transplants [64], and remained elevated in the d25h/n organoid group, reflecting reports of circulating GDF15 levels correlating with increased risk of CKD progression [65] (Fig. 3C-E, Additional file 1: Fig. S3). A hypoxic response was evident by upregulation of stress-responsive protein NDRG1 [66], HIF1 target CCN2 [67], and glycolytic enzymes HK1 and HK2, alongside decreased PCNA and phospholipid phosphatase PLPP3 (Fig. 3C-D).

Proteins associated with inflammatory signalling and epithelial stress were elevated in d25h/n injured organoids, including ICAM1, KRT7/8/18/19, APOE, S100A8/9 and CCN1 (Fig. 3E-H and Additional file 1: Fig. S3). Increased ICAM1 and localisation to tubular structures was confirmed by immunofluorescence (Fig. 3G). Markers of cellular and oxidative stress were elevated, including HSPA5 (d20h and d25h/n), SOD2 (d25h/n) (Fig. 3F), together with enrichment of endoplasmic reticulum stress pathways (Additional file 1: Fig. S2D).

Structural changes were also evident in d25h/n organoids, including reduced tubular density, disrupted epithelial morphology, and thickened basement membranes, with increased interstitial staining between tubules, mirroring nephron loss and tissue remodelling following injury (Fig. 3I, Additional file 1: Fig. S4).

Fewer differentially expressed proteins were detected compared to RNAseq, likely reflecting the lower sensitivity and dynamic range of proteomics and the contribution of post-transcriptional regulation. As bulk assays, these RNAseq and proteomic results cannot resolve cell-type-specific sources of a signal and may be influenced by changes in cell-type proportions over time. Nevertheless, several signatures were conserved across gene and protein profiles, including HIF pathway activation, metabolic reprogramming, extracellular matrix (ECM) remodelling at d20, and increased inflammatory signalling and epithelial stress at d25 (Table 1). This cross-platform validation establishes high-confidence markers of hypoxic injury (e.g. GDF15, CCN1, NDRG1, SLC2A1/3, HK2, P4HA1/2; d20) and persistent injury-associated states (LCN2, S100A8/9, MMP7, KRT18; d25). Consistent with nephron injury, genes and proteins associated with podocyte and proximal tubule identity were reduced at both timepoints.

**Table 1:** RNA-protein correlation: Overlap was calculated by identifying genes and proteins with FDR < 0.05 and absolute logFC > 0.58 in both differential datasets. *‘Nephron markers’ based on gene expression in developing human kidney scRNAseq reference data

RNA-Protein correlation: Acute hypoxic response (d20n vs. d20h)	
Annotation	Shared genes/proteins
Hypoxic response (↑d20h)	GDF15, SFN, NDRG1, KDM3A, EIF1, ACBD3
Glycolysis (↑d20h)	SLC2A1, SLC2A3, HK2,
ECM remodelling (↑d20h)	P4HA1, P4HA2, CCN1, IGFBP2, PLOD1
Nephron markers* (↓d20h)	CUBN, LRP2, ENPEP, GPD1 (expressed in PT); AIF1, NPHS2, CLIC5, GOLM1, CRB2 (expressed in Pod)

**RNA-Protein correlation: Injury - recovery (d25n vs. d25h/n)**	
Inflammation (↑d25h/n)	S100A8, S100A9, ICAM1, HLA-B, LGALS3, PYCARD, ANXA3
Tubular de-differentiation and injury (↑d25h/n)	KRT7, KRT8, KRT18, S100A6, MVP, GDF15, CD47
ECM remodelling/fibrosis (↑d25h/n)	GPNMB, LGALS3, VWA1, PRG4
Nephron markers* (↓d25h/n)	SLC27A2, SCRN2, GATM, MT1H (expressed in PT); PTPRO, AIF1, FOXC2 (expressed in Pod)

### scRNAseq defines changes in organoid cellular composition after hypoxic injury

**Fig. 4:**
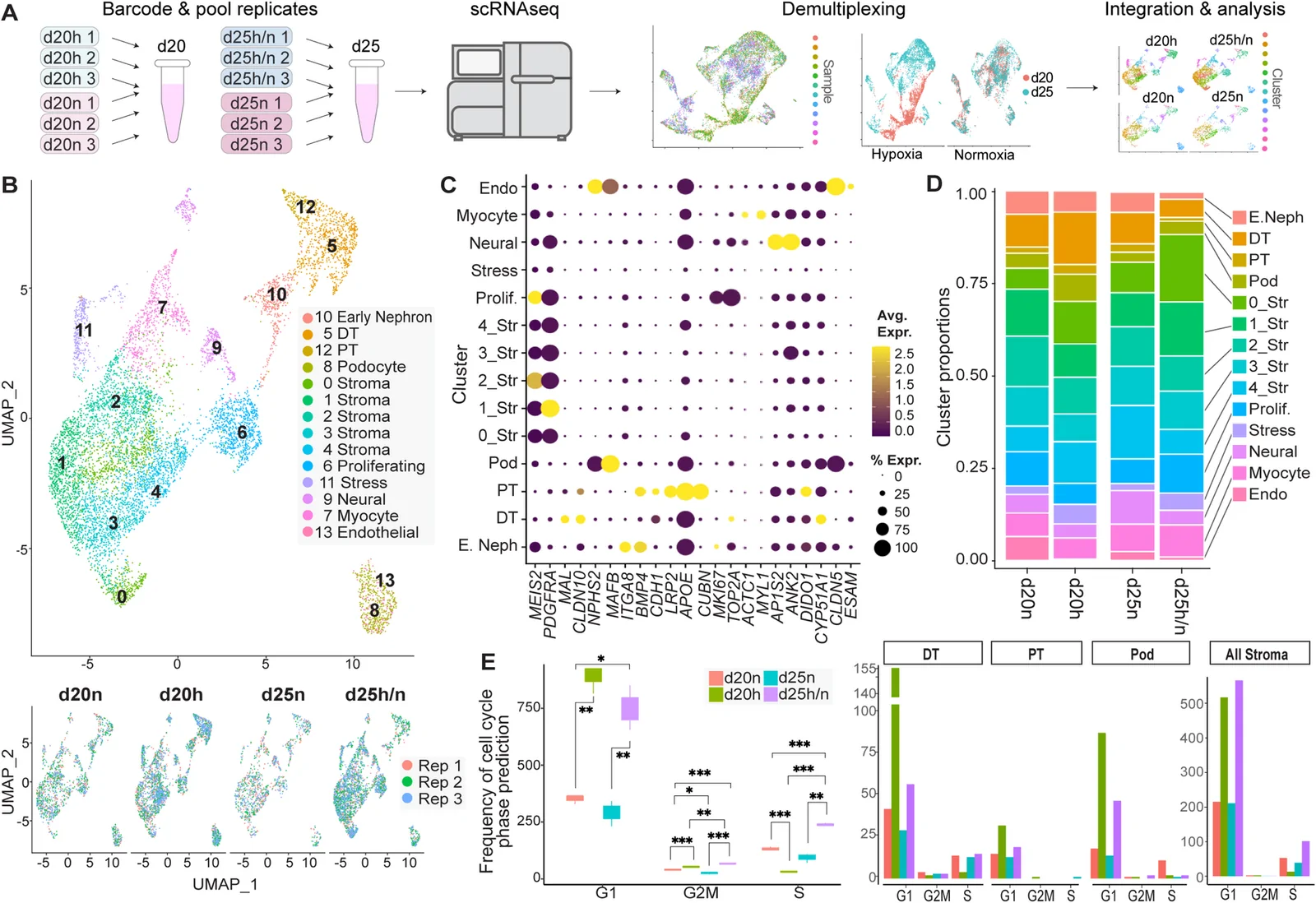
ScRNAseq analysis identifies changes in cell proportions and proliferation after hypoxic injury **A** Overview of scRNAseq experiment. *n* = 3 biological replicates per group, each consist of a pool of 3 organoids; all organoids derived from a single differentiation **B **UMAP plot of integrated d20n, d20h, d25n and d25h/n organoid datasets coloured by cluster. Below, UMAP showing an even distribution of data from three replicates for each condition **C **Dot plot of markers for each of the 14 clusters: stroma (*MEIS2*,* PDGFRA*), DT (*CLDN10*,* MAL*), podocyte (*NPHS2*,* MAFB*), early nephron (*ITGA8*,* BMP4*), PT (*LRP2*,* APOE*,* CUBN*), ‘proliferating’ (*MKI67*,* TOP2A*), ‘cellular stress’ (*DIDO1*,* CYP51A1*), muscle-like (*ACTC1*,* MYL1*) and neural (*AP1S2*,* ANK2*) and endothelial (*CLDN5*,* ESAM*) **D** Bar plot illustrating average proportions of clusters across replicates and conditions. **E** Box plot illustrating the frequency of cells in G1, G2/M and S phase of the cell cycle across all clusters (left), and within DT, PT, Pod and stromal clusters (right), *p < 0.05, **p < 0.01, ***p < 0.001

We next used single cell RNA sequencing (scRNAseq) to investigate changes in cell proportions and injury responses at cellular resolution. Replicate samples from each experimental group were multiplexed using lipid-oligo barcodes and profiled on the 10x Chromium platform (Fig. 4A). Cell populations corresponding to nephron, endothelial, stromal, and off-target lineages were identified based on canonical marker gene expression [48, 68] and annotated using the DEVKidCC classifier [69], a reference-based tool trained on human developmental kidney datasets to assign cell identities in scRNA-seq data. This approach enabled consistent and unbiased classification of organoid cell types within a developmental context (Fig. 4B-C, Additional file 4).

Cell proportion analysis [70] revealed a reduction in nephron progenitor and differentiated nephron cell types after hypoxic injury (30% d20h to 11% d25h/n), alongside expansion of stromal cluster 0 (8% d25n control vs. 18% d25h/n), which is characterised by expression of extracellular matrix and stromal-associated genes *SCRG1*,* SFRP2*,* KAZN* and *SCX*, (Fig. 4D, Additional file 1: Tables S1-S2). Cell cycle analysis revealed that d20h organoids had more cells in G1 and fewer in S-phase, consistent with cell cycle arrest, together with a modest increase in G2/M-phase (Fig. 4E). By d25, injured organoids remained G1-enriched but showed a relative increase in S-phase cells (Fig. 4E), consistent with persistent epithelial stress and proliferative responses reported in AKI [71, 72].

### Divergent epithelial responses and shared inflammatory pathways following hypoxic injury

**Fig. 5:**
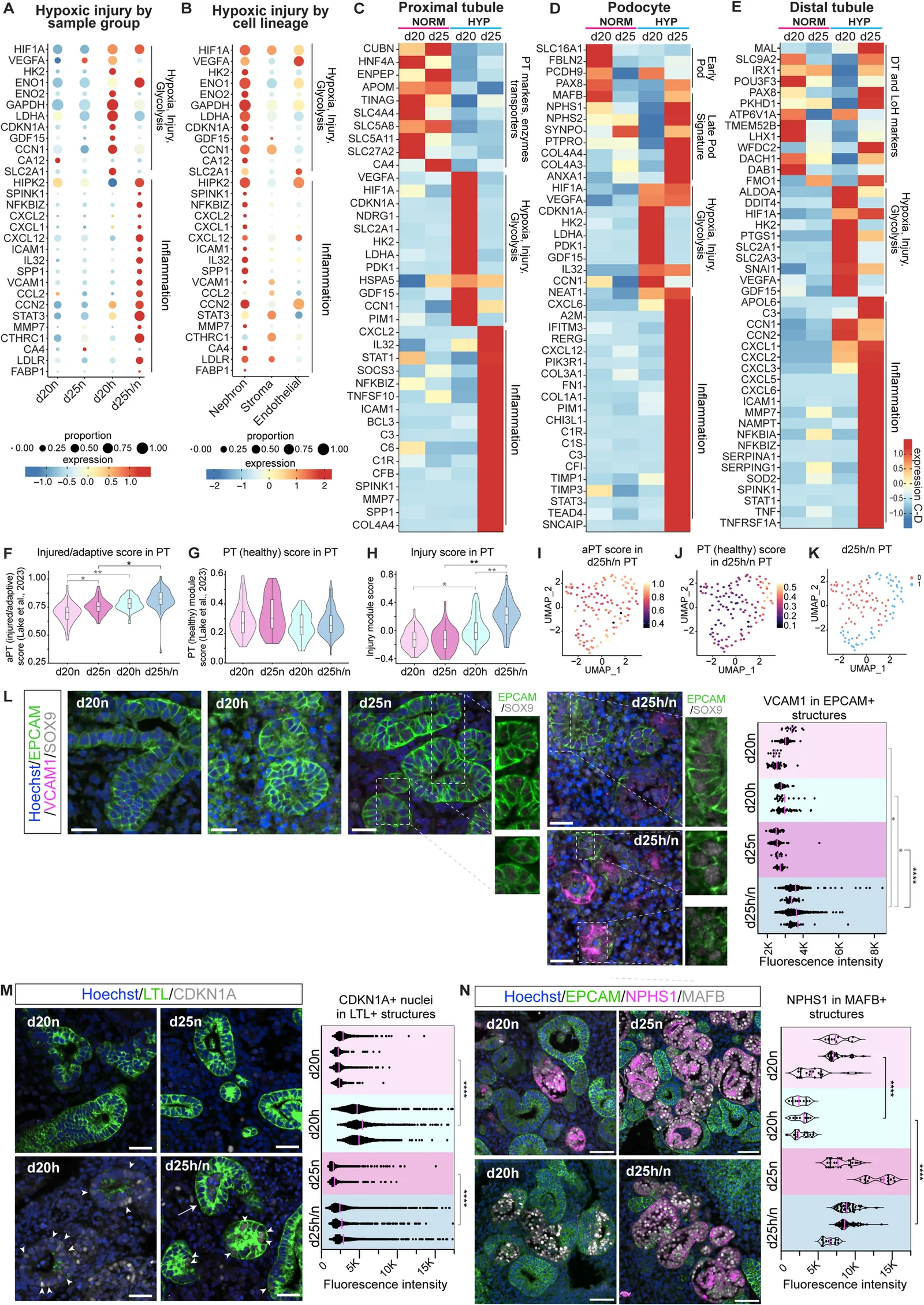
Injury and inflammation signatures are enriched in the nephron, with persistent effects in the proximal tubule **A**-**K** show data derived from *n* = 3 biological replicates as per Fig. 4**A** Dot plot of hypoxia-, injury-, glycolysis-, and inflammation–related genes across all organoid groups shows that hypoxic responses are specific to d20h, while inflammatory programs are restricted to d25h/n **B** Dot plot of hypoxia, injury and inflammation signatures show enrichment in the nephron, compared to stromal and endothelial cell lineages, using expression data aggregated across all organoid groups **C-E** Heat maps of selected differentially expressed genes in proximal tubule, podocyte, and distal tubule scRNA-seq clusters, including cell type markers and genes related to hypoxia, injury, glycolysis, and inflammation. Normalised expression values were scaled by each gene’s mean and standard deviation. Genes plotted have an absolute logFC > 1 and FDR < 0.05 in the d20 or d25 comparisons, except IRX1 and POU3F3, which meet an absolute logFC > 0.58 and FDR < 0.05 **F-H** Module scores for gene sets across time points and treatment groups within the PT cluster. Outlines show the distribution of scores across all cells in each group; boxes represent the interquartile range (25th–75th percentile) with the median indicated, whiskers extend to 1.5× the interquartile range. Statistical differences assessed using two-tailed Welch’s t-tests on replicate means (*n* = 3 organoids per group). Non-significant (ns) comparisons are not shown. Specific p-values are as follows: F (d20n-d25n, 0.0316;d20n-d20h, 0.0056; d25n-d25h/n, 0.0159); H (d20n–d20h, 0.0168; d20h–d25h/n, 0.0058; d25n–d25h/n, 0.0025) **I-J** UMAP visualisations of KPMP adaptive PT (aPT) and healthy PT module scores within the PT cluster **K** UMAP of d25h/n PT subclusters **L** Representative images of VCAM1 and SOX9 staining across organoid groups show an overall increase in VCAM1 levels in d25h/n tubules, with stronger expression in a subset. Increases in SOX9 levels in d25h/n were not statistically significant. Scale bars, 20 μm. Scatterplot of VCAM1 intensity in EPCAM+ structures shows individual values and line at mean. Differences between groups were assessed using two-tailed Welch’s t-tests on replicate means (*n* = 3–4 organoids per group, all derived from one differentiation); non-significant comparisons are not shown. Specific p values: d20n-d25h/n, 0.0179; d20h-d25h/n, 0.0179; d25n-d25h/n, 0.0001 **M** Representative images of CDKN1A staining show increased expression in d20h and d25h/n tubules. Scale bars, 30 μm. Scatterplot of nuclear CDKN1A levels in LTL+ tubules shows individual values and line at mean. Differences were assessed using two-tailed Welch’s t-tests on replicate means (*n* = 2–4 organoids per group, all derived from one differentiation), non-significant comparisons are not shown. Specific p values: d20n-d20h, 0.0006; d25n-d25h/n, 0.0086; not shown on graph: d20n-d25n, 0.0072, d20h-d25h/n, 0.0020. **N** Representative images of MAFB and NPHS1 staining show loss of NPHS1 in d20h and recovery in d25h/n. Scale bars, 50 μm. Violin plot of NPHS1 levels in MAFB^+^NPHS1^+^ structures shows individual values and line at median. Differences were assessed using two-tailed Welch’s t-tests on replicate means (*n* = 2–3 organoids per group, all derived from one differentiation), non-significant comparisons not shown. Specific p values: (d20n-d20h, 0.0331; d20h-d25h/n, 0.0099)

scRNAseq analysis across all experimental groups identified hypoxia-responsive and glycolytic gene expression (e.g. *HIF1A*,* VEGFA*,* HK2*), specifically enriched in d20h organoids, while inflammatory programs were enriched in the d25h/n injury-recovery group (Fig. 5A). These responses were most pronounced within nephron epithelial populations compared to stromal and endothelial compartments, consistent with the primary impact of ischaemic injury on the tubular epithelium (Fig. 5B).

In normoxic controls, nephron cell types exhibited expected expression and maturation of lineage-specific markers between d20n and d25n (Fig. 5C-E, Additional file 4). Proximal tubule, podocyte, and distal tubule markers were largely maintained or increased over time, consistent with ongoing differentiation, although some markers reached peak expression by d20, indicating that key aspects of cell identity are already established by this stage. Within the podocyte population, markers associated with early identity during human kidney development were enriched in d20n (e.g. *SLC16A1*,*PCDH9*,* PAX8*) [73], while markers of later differentiation (e.g. *NPHS2*,* SYNPO*,* PTPRO*,* COL4A3/4*) [73], increased from d20n to d25n, indicating progression towards a more mature state (Fig. 5D).

Hypoxic exposure (d20h) resulted in a broad reduction of cell type–specific and functional gene expression across nephron segments, including proximal tubule regulators and transporters (e.g. *HNF4A*,* CUBN*, SLC family genes), podocyte structural components, and distal tubule transport and polarity markers (Fig. 5C–E). This was accompanied by induction of hypoxia-responsive and glycolytic pathways (*HIF1A*,* HK2*,* LDHA*,* PDK1*,* SLC2A1*) across all epithelial clusters.

Following return to normoxia, divergent recovery responses were observed. Podocyte and distal tubule markers were largely restored in d25h/n organoids, approaching or exceeding mRNA levels observed in d25n controls. In contrast, proximal tubule markers showed only partial recovery, remaining reduced relative to controls, particularly for transport and metabolic genes (Fig. 5C).

Injury-associated and inflammatory transcriptional signatures persisted at d25h/n across all nephron cell types, including upregulation of *GDF15*,* MMP7*,* SPP1*,* CXCL2*,* ICAM1*, and complement components (Fig. 5C-E). TNF-NFκB, NOD-like receptor signalling, Lipid and atherosclerosis, and complement and coagulation cascade pathway signatures were enriched in all segments; JAK–STAT signalling was uniquely enriched in the proximal tubule (Additional file 4). These data indicate that while epithelial differentiation programs can recover in a segment-specific manner, inflammatory and stress-associated transcriptional states persist following hypoxic injury, with the proximal tubule exhibiting the most sustained disruption of epithelial identity.

### Loss of proximal tubule identity and emergence of injury-associated epithelial states

We reasoned that reduced proximal tubule (PT) marker expression following injury may obscure cell identity in unsupervised clustering. To address this, we re-evaluated PT identity using module scoring of established markers. This analysis identified an expanded PT population in d25h/n organoids, with most additional cells originating from the distal tubule cluster (Additional file 1: Fig. S5), indicating a shift away from a canonical PT profile following injury.

To determine whether these changes reflect injury-associated epithelial states observed in human kidney disease, we calculated gene set scores for healthy and adaptive/maladaptive signatures derived from the Kidney Precision Medicine Project (KPMP) [19]. At the cluster level, proximal tubule cells in injured d25h/n organoids showed significant enrichment of KPMP adaptive/maladaptive PT signatures and a custom injury module score (*SOX9*,* CDKN1A*,* VCAM1*,* SPP1*,* PROM1*,* ICAM1*) compared to d25n controls, while ‘healthy’ KPMP PT signatures were not significantly different (Fig. 5F-J). At d25, both the adaptive/maladaptive PT signature and the custom injury score were enriched in hypoxia-exposed organoids relative to stage-matched controls, with the adaptive/maladaptive signature also showing an increase with maturation (Fig. 5F, H).

In reclassified PT cells at day 25, hypoxic injury induced a transcriptional programme consistent with injury-associated proximal tubule states described in human AKI-to-CKD transition datasets (Additional file 4). The most strongly upregulated genes included CXC chemokines - *CXCL2* (Log_2_FC + 6.84), *CXCL5* (+ 6.08), *CXCL6* (+ 5.52), and *CXCL1* (+ 5.34) - alongside *ANKRD1* (+ 5.72) and *ANXA1* (+ 5.58), forming a prominent inflammatory secretory signature aligned with human non-cycling epithelial injury states [23, 24, 26] originally reported in murine ischaemia–reperfusion injury models [11, 15]. *SPP1* was among the most robustly upregulated genes (+ 2.65, FDR 1.4 × 10⁻⁴⁰), consistent with its identification in human injured and adaptive epithelial states [19, 24]. *ICAM1* was also increased (+ 2.95, FDR 6.7 × 10⁻³), aligning with inflammatory proximal tubule phenotypes, including VCAM1⁺/ICAM1⁺ epithelial states described in human kidney disease [24]. Additional stress and inflammatory genes were elevated, including *SPINK1* (+ 5.34) and *STAT1* (+ 1.99). Senescence-associated markers showed modest or inconsistent changes at the cluster level, with *IGFBP7*, *TP53*, and *BCL2* trending upward without significance. Together, these data indicate establishment of inflammatory injury programmes, with features overlapping reported failed repair and maladaptive proximal tubule signatures, without definitive evidence of an established senescence-associated state.

To further resolve heterogeneity within the PT, we reclustered d25h/n PT cells, identifying two subpopulations (Fig. 5K). One was enriched for genes associated with epithelial polarity, adhesion, and developmental signalling, consistent with injury-associated remodelling (cluster 0), while the other was enriched for oxidative phosphorylation and ribosome biogenesis, and showed a higher ‘healthy’ KPMP PT module score (adj.*p* = 1.13 × 10^− 3^, cluster 1) (Fig. 5J, K; Additional file 4). Together, these profiles suggest divergent epithelial responses following injury, spanning adaptive and injury-associated states.

Based on these transcriptional signatures, we next assessed expression of key markers associated with persistent tubular injury - CDKN1A [74], VCAM1 [11, 15], and SOX9 [75], to evaluate protein levels and spatial distribution within tubular structures. CDKN1A and VCAM1 levels were significantly increased in tubular structures following hypoxia, with spatially heterogeneous expression across tubules in d25h/n (Fig. 5L, M, Additional file 1: Fig. S6). SOX9 showed a trend toward increased expression but did not reach significance. EPCAM staining revealed regions of epithelial disruption characterised by diffuse and disorganised signal in d20h organoids, with residual areas of damaged epithelium persisting in d25h/n organoids (Additional file 1: Fig. S6, S7). Across organoids, tubules with low VCAM1 or CDKN1A expression - suggestive of adaptive or effective repair- were observed alongside tubules with high VCAM1 or more CDKN1A^+^ cells, consistent with injured states linked to persistent dysfunction in human kidney disease (Fig. 5L, M, Additional file 1: Fig. S6). These findings support a model in which hypoxic injury induces loss of PT identity and emergence of injury-associated epithelial states, rather than simple depletion of PT cells.

A subset of podocyte markers showed reduced transcript abundance between d20n and d25n in scRNAseq analysis, inconsistent with the expected trajectory of increasing maturation. To investigate this, we quantified protein expression within NPHS1⁺/MAFB⁺ structures (Fig. 5N, Additional file 1: Fig. S7). NPHS1 protein levels were reduced following hypoxic exposure (d20h) and recovered in d25h/n organoids to levels comparable to d25n controls. NPHS1 protein expression was maintained or increased between d20n and d25n, while MAFB levels were stable across all conditions. Together, these data support continued podocyte maturation in normoxic organoids and indicate that reductions in transcript abundance do not reflect loss of podocyte identity. Importantly, the reduction of NPHS1 within established NPHS1⁺/MAFB⁺ structures at d20h supports injury to differentiated nephron segments rather than a failure of differentiation. Consistent with this, marker expression in d25h/n podocytes was biased towards maturing podocyte gene signatures (Fig. 5D).

### Hypoxia induces metabolic reprogramming and persistent metabolic dysregulation in kidney organoids

**Fig. 6:**
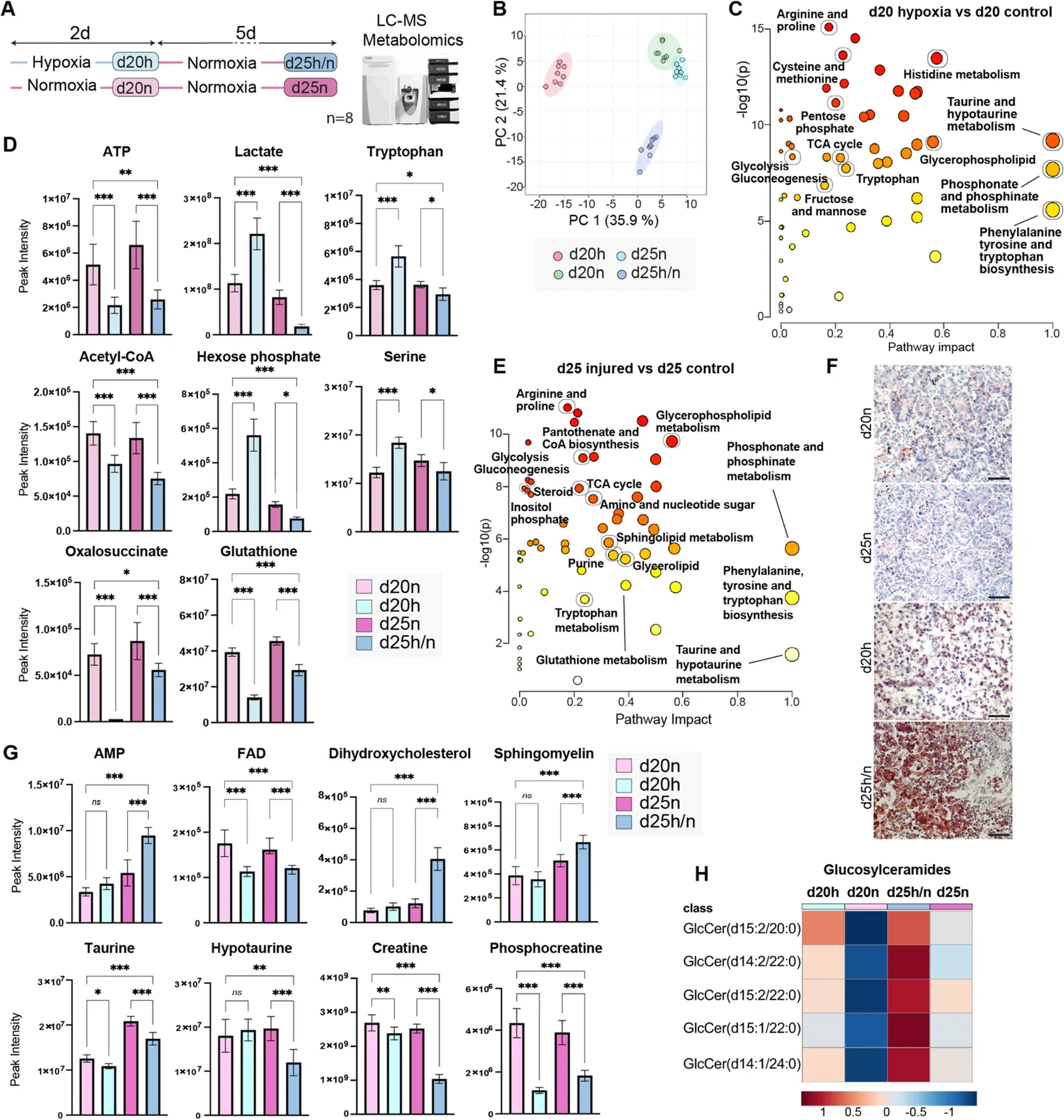
MS-based metabolomics supports metabolic dysregulation and mitochondrial dysfunction during and after hypoxic injury** A** Overview of metabolomic experiment with 8 organoids per condition, all derived from one differentiation **B** PCA analysis of metabolomic data, which were normalized by the median, log10 transformed, and pareto scaled **C** Metabolic pathway enrichment analysis of differences between d20h and d20n organoids. Circle size represents pathway impact score (x-axis); y-axis indicates significance, with stronger *p-*values depicted in red. Select pathways related to Ischaemic AKI are outlined and labelled **D** Peak intensity of select metabolites dysregulated after hypoxia. Statistical comparisons utilised two-tailed Welch’s t-tests on replicate means *p < 0.05, **p < 0.01, ***p < 0.001 **E** Pathway enrichment analysis between d25h/n and d25n organoids. Select pathways related to AKI-CKD transition are outlined and labelled **F** Oil Red O staining of control and injured organoids at d20 and d25. Images representative of an experiment containing *n* = 4 organoids per group, all derived from one differentiation. Scale bars, 50 μm **G** Peak intensity of select CKD-associated metabolites in d25 injured organoids. Statistical comparisons utilised two-tailed Welch’s t-tests on replicate means *p < 0.05, **p < 0.01, ***p < 0.001 **H** Heatmap showing five different glucosylceramide (GlcCer) species increased in d25h/n organoids

Our data suggest that hypoxic exposure triggers immediate (d20) and longer term (d25) changes in metabolism. We interrogated these changes with untargeted LC-MS metabolomics on 8 organoids per group (Fig. 6A-B; Additional file 5). Profiles were highly conserved within groups, while substantial differences were observed between injured organoids and controls (Fig. 6B).

Pathway enrichment analysis of metabolites in d20 hypoxic and d20 control organoids indicated dysregulation of arginine and proline metabolism, cysteine and methionine metabolism, the pentose phosphate pathway, tricarboxylic acid (TCA) cycle, tryptophan metabolism, and glycolysis/gluconeogenesis (Fig. 6C). Levels of ATP were decreased while lactate levels were increased in d20h organoids, consistent with a shift toward glycolysis (Fig. 6D). TCA-associated metabolites acetyl-CoA and oxalosuccinate were decreased in d20h organoids (Fig. 6D) indicating reduced carbon flux into the TCA cycle during prolonged hypoxia [76]. Levels of tryptophan, serine, and putative hexose-phosphate, were significantly increased in d20h organoids (Fig. 6D), suggesting altered amino acid metabolism in hypoxia [77]. Levels of the antioxidant glutathione were significantly reduced (Fig. 6D).

Unbiased enrichment analysis comparing d25h/n and d25n identified disruptions in multiple metabolic pathways, including arginine and proline, glycerolipid and glycerophospholipid, sphingolipid, steroid, tryptophan, and TCA metabolism, as well as glycolysis/gluconeogenesis (Fig. 6E). Consistent with these findings, Oil Red O staining revealed neutral lipid accumulation, particularly in tubular structures of injured organoids (Fig. 6F). Despite 5 days of recovery in normoxic conditions, d25h/n organoids failed to restore ATP levels and instead showed increased AMP (Fig. 6G), resulting in a reduced ATP/AMP ratio indicative of energetic stress [68]. Reduced levels of TCA-associated metabolites (oxalosuccinate, acetyl-CoA, and FAD) further support mitochondrial dysfunction (Fig. 6D and G).

A dyslipidaemia-like phenotype was also evident, with increased oxysterol-related metabolites, associated with oxidative stress and chronic kidney disease, and elevated sphingomyelins and ceramides associated with kidney pathology [78, 79] (Fig. 6G, H). Amino acid metabolism was altered, with significant reductions in taurine, hypotaurine, tryptophan, creatine, and phosphocreatine-metabolites similarly reduced in CKD patients [80–83] (Fig. 6G). Collectively, hypoxia-injured human kidney organoids recapitulate metabolic features of human AKI and injury-associated metabolic reprogramming.

### Hypoxic injury drives macrophage activation in human kidney organoids

**Fig. 7:**
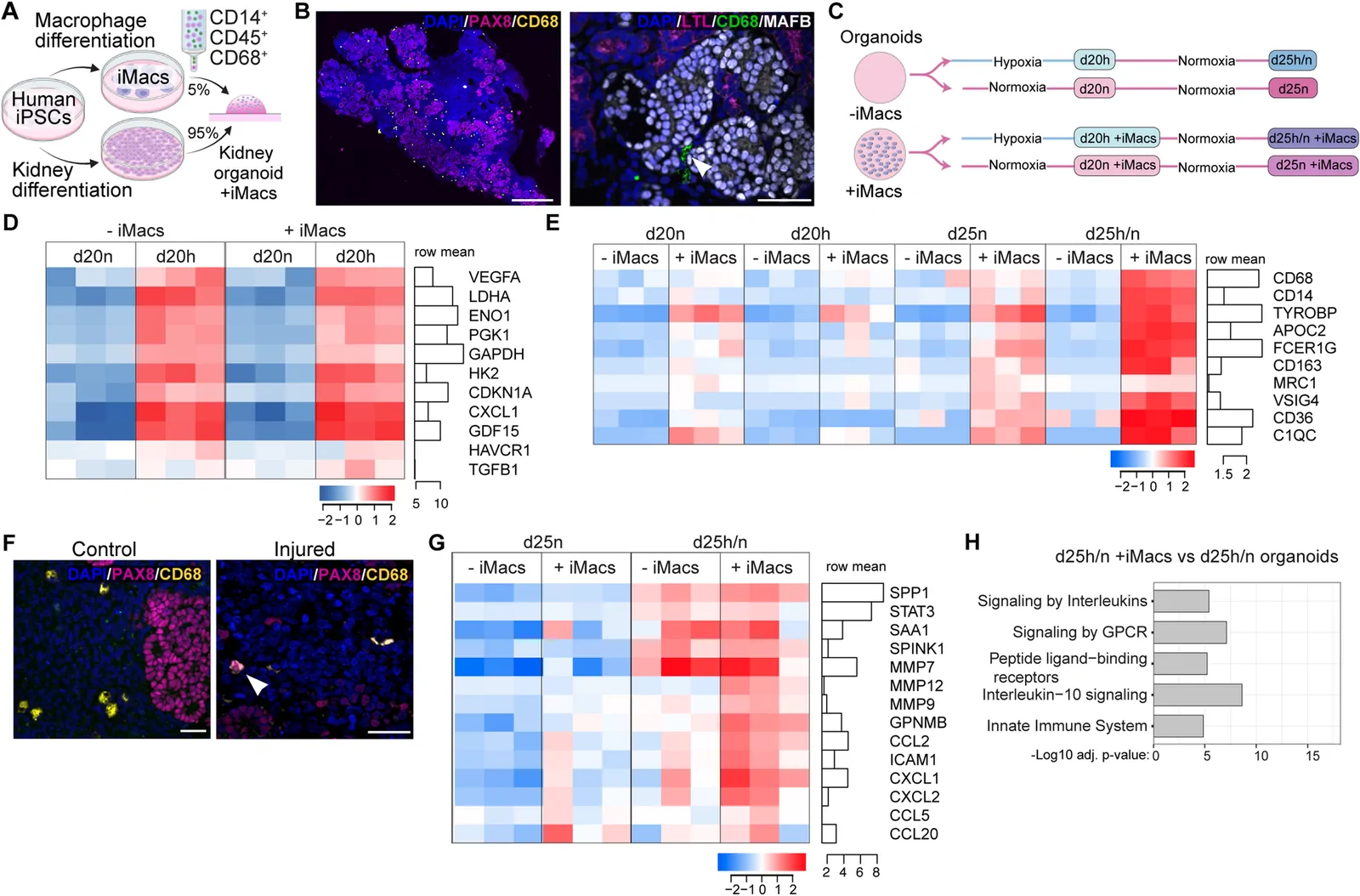
Human macrophages mount a pro-inflammatory response in injured organoids **A** Overview of iMac and kidney organoid differentiation and co-culture **B** Representative images of d20 kidney organoids labelled with markers of the epithelium (PAX8), proximal tubule (LTL), podocytes (MAFB), macrophages (CD68, arrowhead in right image) and nuclei (DAPI). Scale bars: 500 μm left image, 50 μm right image **C** Overview of hypoxic injury experiment organoids +/- macrophages exposed to 48 h of hypoxia or normoxia and harvested at d20 and d25. *n* = 3 organoids per group with all derived from one differentiation **D** Heatmap of hypoxic response and injury markers in d20 organoids (Log2 CPM-normalised expression) **E** Heatmap of macrophage markers in d20 and d25 organoids, illustrating iMac activation in d25h/n injured organoids (Log2 CPM-normalised expression) **F** Representative images of d20 kidney organoids labelled with PAX8 and CD68 showing potential macrophage engulfment of a damaged epithelial cell (arrowhead); Scale bars, 50 μm **G** Heatmap comparing inflammatory markers in d25 organoids (Log2 CPM-normalised expression) **H** Upregulated pathways in d25h/n iMacs-containing injured organoids compared to injured controls (FDR < 0.05)

Immune cell populations play central roles in determining kidney injury outcomes, with macrophages acting as key mediators of repair or progression to chronic disease [17, 18, 84]. To model immune–epithelial interactions during kidney injury, human iPSC-derived macrophages (iMacs) were differentiated using an established protocol [38, 40, 41] yielding cells with macrophage morphology and phagocytic activity [38], and a transcriptional profile resembling yolk sac-derived tissue-resident macrophages rather than circulating monocytes or dendritic cells [40]. iMacs were incorporated into kidney organoids at day 7 of differentiation, and their presence was confirmed by CD68 staining at days 20 and 25 (Fig. 7A-C). Bulk RNA sequencing of iMac-containing and control organoids cultured under normoxic or hypoxic conditions revealed no major differences in hypoxic response or injury-associated gene expression (Fig. 7D, Additional file 1: Fig. S8, Additional file 6).

Differential expression analysis of iMac-containing organoids under normoxic conditions revealed significant upregulation of macrophage-associated markers *CD68*,* MRC1*,* CSF1R*,* FCGR1A*, compared to normoxic organoids lacking iMacs. *VSIG4*,* C1QC*, and *MS4A* family genes were significantly increased, consistent with differentiated and tissue-resident macrophage transcriptional programs that are typically enriched in macrophages relative to circulating monocytes or immature myeloid precursors (Fig. 7E, Additional file 6). No established markers of macrophage polarization or kidney cell types were differentially expressed, suggesting that iMacs adopt a homeostatic, resident-like phenotype and that incorporation of 5% iMacs at day 7 does not substantially alter kidney organoid differentiation.

Following hypoxic injury, d25h/n iMac-containing organoids showed a significant increase in macrophage activation markers (Fig. 7E) without a change in macrophage numbers (Additional file 1: Fig. S8). Immunofluorescence revealed PAX8 internalization in CD68⁺ iMacs, consistent with phagocytosis of injured epithelial cells (Fig. 7F). Injury-associated epithelial markers (*SPP1*,* STAT3*,* SAA1*,* SPINK1*,* MMP7*) remained elevated in injured iMac-containing organoids, alongside higher *MMP9*,* MMP12* and *GPNMB*, which are expressed in activated macrophages. Pro-inflammatory cytokines and chemokines *CCL2*,* CCL5*,* CCL20*,* CXCL1/2* and *ICAM1* were also upregulated, with enrichment of innate immune and interleukin signaling pathways (Fig. 7G, H). Together, these data suggest that iMacs mount a pro-inflammatory response in hypoxia-injured kidney organoids, recapitulating key features of macrophage activation during AKI.

### Spatial transcriptomic analysis of macrophage influence on injury and repair

**Fig. 8:**
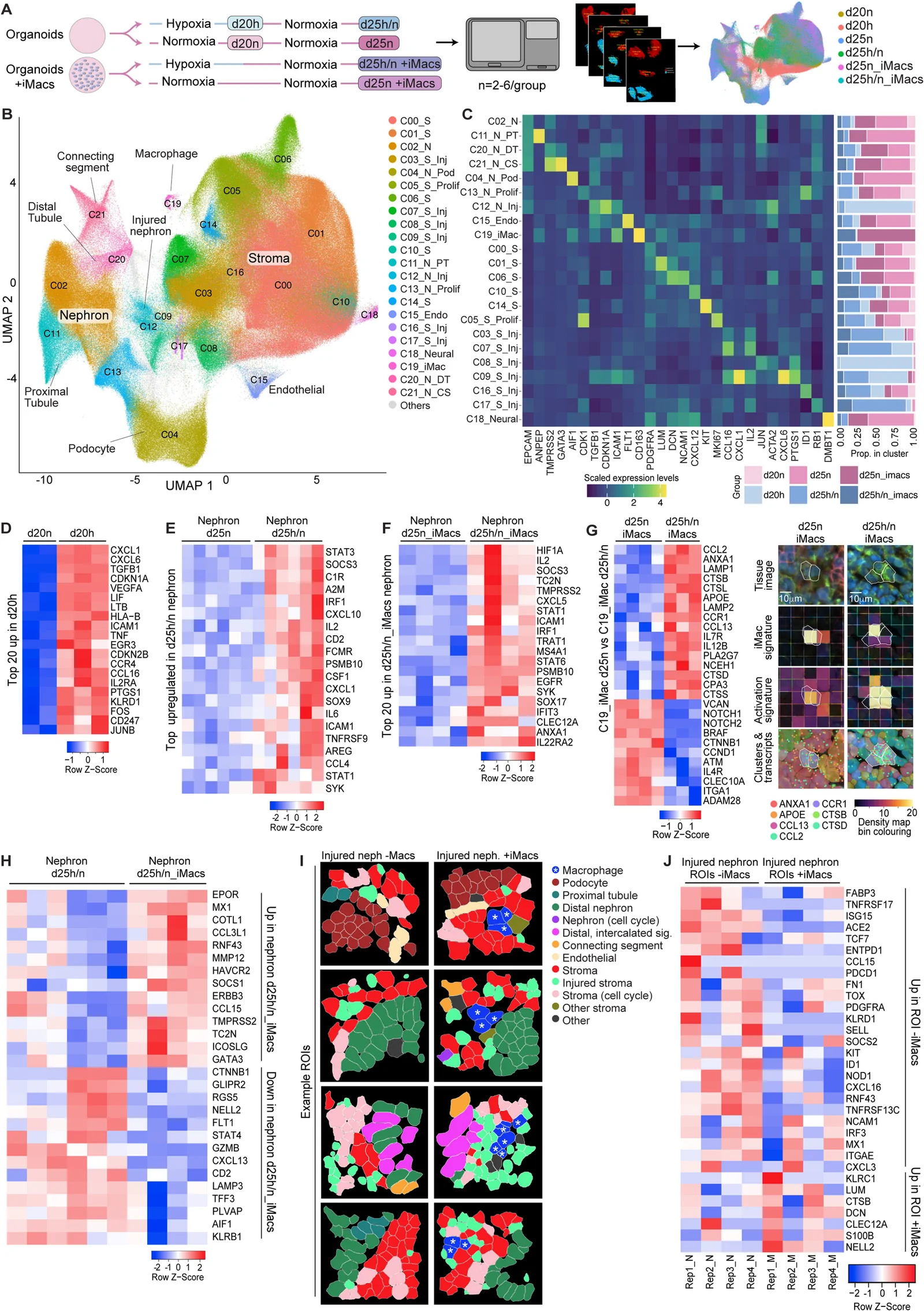
Spatial transcriptomic analysis of macrophage influence on injury and repair** A** Experimental overview. *n* = 2-6 organoids per experimental group (d20n, *n* = 2; d20h, *n* = 3; d25n, *n* = 6; d25h/n, *n* = 6 d25n+iMacs, *n* = 4; d25h/n+iMacs, *n* = 4; all organoids derived from one differentiation aside from d25n and d25h/n, which were derived from two batches as defined in Methods **B** UMAP of clusters from Xenium spatial data **C** Heatmap showing key marker genes supporting cluster identity (left) and proportions of cells within a cluster, coloured by sample group (right) **D** Heatmap of top upregulated genes in d20h organoid spatial data compared to d20n supporting a hypoxic injury signature **E** Heatmap of top upregulated genes in the nephron lineage of d25h/n organoid spatial data compared to d25n supporting persistent injury and inflammation **F** Heatmap of top upregulated genes in the nephron lineage of d25h/n_iMacs organoid spatial data compared to d25n_iMacs, supporting persistent injury and inflammation **G** Left - Heatmap of top up and downregulated genes in the macrophage cluster (C19) after hypoxic injury at d25, showing activation of inflammatory and phagocytic markers after injury. Right- representative regions of interest containing macrophages in d25n_iMacs and d25h_iMacs organoids showing tissue image (iMacs outlined), ‘iMac signature’ (density map for macrophage markers *CD14*,* CD68*,* PTPRC*), ‘activation signature’ (density map of select genes upregulated in injured organoids *ANXA1*,* APOE*,* CCL13*,* CCL2*,* CCR1*,* CTSB*,* CTSD*), cluster allocations and ‘activation signature’ transcripts **H** Heatmap of top differentially expressed genes between injured nephron clusters in organoids with or without iMacs **I** Representative regions of interest (ROIs) containing injured nephrons, that have macrophages in the area, and comparable regions that lack macrophages. All ROIs are from d25h/n_imacs organoids **J** Heatmap of differentially expressed genes upregulated in injured nephron regions containing or lacking macrophages. FDR and LogFC values for analysis in this figure are available in Additional files 7-9. Columns show replicate samples in heatmaps D-H, columns show average of ROIs within a sample for J

To further interrogate how macrophages respond to hypoxia and affect the injury response at a cellular level, we profiled injured iMac-containing organoids and controls using the Xenium Human Immuno-Oncology (I/O) panel. The combined dataset contained 835,557 cells from 25 organoids and 6 experimental groups (Fig. 8A, Additional file 1: Fig. S9). Clustering and scRNAseq-guided annotation of the Xenium data defined expected nephron, stromal, endothelial and macrophage cell types (Fig. 8B, C, Additional file 7). Cell type identities were assigned using unsupervised clustering, integration with reference human kidney organoid scRNA-seq data (label transfer and marker gene comparison), and spatial validation of marker expression patterns against tissue morphology. This combined approach enabled confident identification of nephron subtypes, including distal and connecting segment clusters (C02, C13, C20, C21), proximal tubule (C11), podocyte (C04) and an injured epithelial population (C12), marked by *EPCAM*, *TGFB1*,* CDKN1A*, *ICAM1*, *APOE* and *ACTA2* (Fig. 8C, Additional file 7). The injured epithelial cluster (C12) was almost exclusively derived from hypoxia-exposed conditions (> 90%), whereas multiple stromal clusters displayed a similar pattern, indicating stromal injury-associated states (Fig. 8C).

Differential expression analyses of Xenium data captured both acute (d20) hypoxia-responsive and persistent (d25) injury-associated transcriptional programmes (Fig. 8D-F, Additional file 8), consistent with observations from bulk and single-cell analyses, confirming effective induction of hypoxic injury in this platform. This enabled analysis of iMac incorporation, with comparison of nephron clusters in d25 injured and control organoids revealing enrichment of inflammatory signalling and injury-associated epithelial states across conditions with and without iMacs (Fig. 8E-F).

To confirm macrophage identity, we examined iMac transcriptional profiles at single-cell resolution in the Xenium data. Cluster-level expression of canonical macrophage markers (*CSF1R*,* CD68*,* MPEG1*,* AIF1*,* FCGR1A*), along with resident macrophage–associated genes (*VSIG4*,* C1QB*,* APOE*,* TREM2*,* CD163*), supports a differentiated tissue-resident macrophage identity (Additional file 7).

We next assessed how iMacs respond to hypoxia. Differential expression analysis comparing iMac clusters from d25 control and hypoxia-injured organoids indicates a shift from a proliferative, tissue-remodeling phenotype under normoxic conditions to an activated inflammatory state following hypoxic injury. iMacs in hypoxia-injured organoids upregulated chemokines and inflammatory mediators (*CCL2*,* CCL13*,* CCR1*,* IL12B*), lipid metabolism and damage-response genes (*APOE*,* NCEH1*,* PLA2G7*,* ANXA1*), lysosomal proteases and membrane components (*CTSB*,* CTSL*,* CTSS*,* CTSD*,* LAMP1*,* LAMP2*), consistent with enhanced phagolysosomal activity and activation of chemokine signalling associated with immune cell recruitment. In contrast, iMacs from control organoids showed higher expression of genes associated with proliferation, developmental signaling, and extracellular matrix remodeling (*CTNNB1*,* BRAF*,* CCND1*,* NOTCH1*,* NOTCH2*,* VCAN*,* ITGA1*), along with receptors linked to homeostatic immune signaling (*IL4R*,* CLEC10A*), indicating a homeostatic or reparative state under normoxic conditions (Fig. 8G, Additional file 8).

To assess the impact of iMacs on epithelial injury responses, we compared nephron clusters from injured organoids with and without iMacs. Nephrons in iMac-containing organoids expressed a broader range of ECM-remodelling (*SDC1*,* MMP12*), immune recruitment (*CCL15*,* CCL3L1*,* ICOSLG*), and immune regulatory factors (*SOCS1*,* ICOSLG*,* MX1)*, indicating macrophage-associated effects on epithelial injury responses (Fig. 8H). In contrast, injured nephrons from organoids lacking iMacs upregulated alternative programs, including immune recruitment (*CXCL13*,* CD2*,* KLRB1*), epithelial plasticity (*CTNNB1*), trafficking (*LAMP3*), and endothelial-associated (*FLT1*,* PLVAP*) genes, suggesting divergent epithelial responses in the absence of macrophage input (Fig. 8H).

We next examined macrophage effects within local tissue niches by comparing injured nephron regions with and without iMacs in the same organoids (Fig. 8I, J; Additional file 9). Regions containing injured nephrons and iMacs showed upregulation of macrophage activation, phagocytic and immunomodulatory genes, indicating an activated macrophage niche engaged in phagocytosis, cytokine signaling, and immune regulation. In contrast, injured regions lacking iMacs expressed genes associated with proliferation, survival, and repair-associated programs (Additional file 9). Nephron epithelial cells within iMac-containing regions exhibited a mixed profile spanning proteolysis and injury responses (*CTSB*), matrix organization (*DCN*,* LUM*), immune regulation (*KLRC1*,* CLEC12A*), and stress signalling (*S100B*,* NELL2*) (Fig. 8J). Regions lacking iMacs show related but distinct profiles, including fibrosis-associated genes (*FN1*,* PDGFRA*), differentiation and stress responses (*ID1*,* RNF43*,* FABP3*,* TOX*), and chemokine signalling (*CXCL3*,* CXCL16*,* CCL15*) (Fig. 8J).

Despite limitations of the spatial analysis, including the targeted gene panel, variability in sectioning, and the low abundance of iMacs following injury, these data show that iMacs respond to hypoxic injury by adopting an inflammatory, lysosome-active state. They are also associated with modulation of local epithelial injury responses through phagocytic, immune regulatory, and matrix-remodelling programs.

## Discussion

Ischaemia is a leading cause of AKI and a driver of progression to chronic kidney disease [5], yet mechanistic studies in humans are limited by inter-patient variability and restricted access to tissue. This highlights a need for scalable human model systems that complement existing in vivo approaches and enable controlled, high-throughput interrogation of injury responses and therapeutic targets. Here, we show that iPSC-derived human kidney organoids recapitulate key epithelial responses to hypoxic injury, including metabolic reprogramming, sustained inflammatory signalling and heterogeneous epithelial recovery. We further show that tissue-resident-like macrophages integrated into kidney organoids adopt activated, lysosome-enriched inflammatory states following injury. Together, these findings position kidney organoids as a tractable human platform to study ischaemic injury-associated epithelial states and immune-mediated repair in AKI.

Hypoxic injury elicited acute epithelial stress responses characterised by HIF activation, glycolytic reprogramming, cell cycle arrest, and reduced expression of cell type-specific markers, consistent with canonical injury responses. These changes were accompanied by rapid metabolic rewiring, including increased lactate, reduced ATP, and depletion of TCA cycle intermediates, indicating a shift toward glycolysis and impaired mitochondrial metabolism under hypoxia. Following return to normoxia, metabolic recovery was incomplete, with persistent energetic stress marked by reduced ATP/AMP ratios and depletion of TCA-associated metabolites, consistent with ongoing mitochondrial dysfunction. Across transcriptomic and proteomic datasets, numerous markers linked to clinical AKI and chronic kidney disease were upregulated (Additional file 1: Table S3). Metabolomic profiling further revealed sustained dysregulation of glycolysis/gluconeogenesis, amino acid metabolism, and lipid pathways, including accumulation of sphingolipids and sterol-related metabolites and neutral lipid deposition within tubular structures, highlighting coordinated metabolic and inflammatory disruption. The requirement for prolonged hypoxic exposure to induce sustained injury responses likely reflects the metabolic conditions used for these organoid cultures, including high glucose concentrations and other exogenous nutrient sources that may partially buffer the energetic consequences of oxygen deprivation.

At single-cell resolution, injury-associated and inflammatory transcriptional programmes persisted across all nephron cell types, including upregulation of GDF15, MMP7, SPP1, CXCL2, and ICAM1, and enrichment of TNF-NFκB-, lipid-, and complement-associated pathways. Nephron cell type-specific markers and functional genes were downregulated following hypoxia, but their recovery was segment-dependent, with proximal tubules showing sustained disruption relative to distal segments and podocytes, consistent with in vivo susceptibility. Although reduced nephron marker expression could reflect perturbed differentiation, several lines of evidence support an injury-driven model: nephron identities are established prior to injury [57], immunofluorescence demonstrates loss of epithelial and podocyte markers within pre-existing structures, and expression of late-stage podocyte markers in recovering glomeruli indicates ongoing maturation rather than developmental arrest. Injured proximal tubules were enriched for KPMP adaptive/maladaptive signatures [19] and exhibited heterogeneous recovery, with subsets restoring cell-type identity and others retaining dedifferentiated, injury-associated states. Alignment with canonical injury markers was incomplete, with inconsistent induction of KIM1/HAVCR1, lack of sustained SOX9 expression, and variable upregulation of VCAM1 and CDKN1A. This may reflect the developmental setting and limited experimental window of this model, as failed and maladaptive repair states consolidate over weeks following injury in vivo.

Our findings are consistent with a growing body of work using kidney organoids to model acute injury across diverse paradigms, including nephrotoxic [29, 30, 85, 86], oxidative [87], and inflammatory [88] insults. Across these systems, organoids reproducibly show induction of injury markers, inflammatory signalling, and segment-specific vulnerability - particularly within proximal tubules. Notably, extended culture models subjected to prolonged or repeated injury develop features associated with AKI-to-CKD transition, including SOX9 activation, G2/M arrest, and profibrotic pathway activation [30, 89]. In this context, our hypoxia-based model recapitulates key shared features, including inflammatory signalling and heterogeneous epithelial recovery, while lacking fully established maladaptive repair states. These differences likely reflect variation in injury modality, duration, and organoid maturity, and highlight that current organoid systems capture conserved injury-associated responses while incompletely modelling later stages of disease progression.

A key distinction of this model is the incorporation of iPSC-derived macrophages, enabling interrogation of macrophage activation and epithelial-immune interactions following injury. To maintain physiological relevance, macrophages were included at low abundance to approximate in vivo proportions [90] rather than creating a macrophage-enriched system. Spatial transcriptomic analysis was therefore essential to resolve macrophage states, revealing a shift from homeostatic, resident-like profiles to activated phenotypes following injury, with increased expression of cytokines, chemokines, and lysosomal and matrix remodelling factors. While constrained by low macrophage abundance, absence of monocyte recruitment, and the limited breadth of the targeted spatial panel, the system supports modelling of innate immune-epithelial crosstalk, with improved resolution of spatially restricted injury states a focus for future studies.

## Conclusions

Together, our findings demonstrate that human kidney organoids recapitulate key epithelial and immune features of hypoxic injury, including metabolic reprogramming, persistent inflammatory signalling, segment-specific vulnerability, heterogeneous epithelial recovery, and macrophage activation. While developmental immaturity, limited vascularisation, and incomplete immune complexity constrain modelling of chronic and fibrotic outcomes, this system provides a tractable and scalable human platform to investigate injury-associated cell states and epithelial-immune interactions in AKI. Continued refinement of organoid maturation, metabolic conditions, vascular integration, and injury paradigms will further enhance their utility for mechanistic studies and therapeutic discovery in human kidney disease.

## Supplementary Information

Additional file 1: Additional Methods, Fig. S1-S9, Table S1-S3.

Additional file 2: Bulk RNAseq.

Additional file 3: Proteomics.

Additional file 4: scRNAseq.

Additional file 5: Metabolomics

Additional file 6: Bulk RNAseq iMacs.

Additional file 7: Xenium cluster markers.

Additional file 8: Xenium pseudobulk DE.

Additional file 9: Xenium ROI DE.

## Data Availability

The Xenium data used in Fig. 1A is available through Zenodo (10.5281/zenodo.20889605) [91]. Bulk RNA sequencing data are available from NCBI GSE236379 (https://www.ncbi.nlm.nih.gov/geo/query/acc.cgi?acc=GSE236379) [92]. Bulk RNA sequencing data from macrophage co-culture experiments are available under accession GSE278562 (https://www.ncbi.nlm.nih.gov/geo/query/acc.cgi?acc=GSE278562) [93]. Single-cell RNA sequencing data are available under accession GSE242425 (https://www.ncbi.nlm.nih.gov/geo/query/acc.cgi?acc=GSE242425) [94]. Xenium spatial transcriptomic data of control and iMac-integrated organoids under normoxic and hypoxic conditions are available under accession GSE307588 (https://www.ncbi.nlm.nih.gov/geo/query/acc.cgi?acc=GSE307588) [95]. Proteomics data are available via PRIDE (ProteomeXchange Consortium) under accession PXD042882 (https://www.ebi.ac.uk/pride/archive/projects/PXD042882) [96]. Metabolomics data are available through Store. Monash under accession 16985 (https://store.erc.monash.edu.au/experiment/view/16985/) [97]. Source code and analysis workflows are available through GitHub (https://github.com/MonashBioinformaticsPlatform/sc-hyp-org) [98] and archived in Zenodo (https://zenodo.org/records/20840256) [99]. An interactive web application for exploratory analysis of the single-cell data, including cell cluster visualisation, gene expression exploration, and marker gene analysis, is publicly accessible at https://bioinformatics.erc.monash.edu/apps/hypoxia-kidney-organoid/ [100]. Human iPSC lines were obtained under material transfer agreements with CSIRO and MCRI.

## Abbreviations

AKI: Acute kidney injury
CKD: Chronic kidney disease
DE: Differential expression
DT: Distal tubule
ECM: Extracellular matrix
FC: Fold change
FDR: False discovery rate
iMacs: iPSC-derived macrophages
iPSC: Induced pluripotent stem cell
KEGG: Kyoto Encyclopedia of Genes and Genomes
KPMP: Kidney Precision Medicine Project
LC-MS: Liquid chromatography-mass spectrometry
PT: Proximal tubule
qPCR: quantitative PCR
ROIs: Regions of interest
scRNAseq: Single-cell RNA sequencing
TCA: Tricarboxylic acid
UMAP: Uniform Manifold Approximation and Projection
