# Supplementary material for: Modelling ischaemic AKI in human kidney organoids reveals injury-associated epithelial states and macrophage-epithelial crosstalk
Source: Genome Med. 2026 Aug 19;18:119. doi: 10.1186/s13073-026-01712-z (PMC13487967; doi:10.1186/s13073-026-01712-z)
Supplement: Supplementary file 1 [file 13073_2026_1712_MOESM1_ESM.pdf]

Supplementary Document for:

Modelling ischaemic AKI in human kidney organoids reveals injury-associated epithelial states and macrophage–epithelial crosstalk

Nunez-Nescolarde et al.

This file includes:

Supplementary Materials and Methods

Supplementary Figures 1 to 8

Supplementary Tables 1 to 3

Supplementary Materials and Methods

Generation of Kidney Organoids

Human iPSCs were directed to differentiate into kidney organoids using the Takasato protocol (1-3), with minor modifications. iPSCs were seeded on Matrigel-coated 6-well plates (Corning, cat no. 354277) using E8 media (Thermo Fisher Scientific) supplemented with 10 μM of ROCK inhibitor Y-27632 (In Vitro Technologies) and 1% Pen/Strep (Thermo Fisher Scientific). The next day (day 0 of differentiation, d0), media was changed to Essential 6 (E6) (Thermo Fisher Scientific), which was initially supplemented with CHIR99021 (In Vitro Technologies) and 1% Pen/Strep (Thermo Fisher Scientific). On day 4, 200 ng/ml of FGF9 (In Vitro Technologies) and 1 μg/ml of Heparin (Sigma-Aldrich) were added to E6. On d7, cells were dissociated with TrypLE Select Enzyme (1X) (Thermo Fisher Scientific) and 150 000 cells were centrifuged in 1.5 ml tubes to generate each organoid. Newly formed organoids were treated with CHIR99021 in E6 medium for one hour (Supplemental Table S1). Organoids were cultured in E6 supplemented with 200 ng/ml of FGF9, 1 μg/ml of Heparin and 1 % Pen/Strep until d13. After, only E6 and Pen/Strep were used.

Cell seeding density and CHIR99021 concentrations were optimized for each iPSC line as follows. 522.3 and 808.5 lines were seeded at 50,000-80,000 cells per well at d-1, treated with 4 μM CHIR99021 from d0-d4. The D4C4 line was seeded at 70,000-100,000 at d-1 and treated with 6 μM CHIR99021 from d0-d4. Differentiations from all lines were dissociated into single cell suspensions at d7 and 150,000 cells were aggregated by centrifugation to generate organoids. Concentrations of CHIR99021 used to induce nephron formation at d7 were 4 μM (522.3 and 808.5) and 5 μM (D4C4).

Kidney organoid differentiation is associated with inherent variability in the timing of cellular maturation and relative cell-type composition between batches, as well as across iPSC lines and laboratories. Such variation can arise from differences in iPSC epigenetic state, media composition and handling, reagent performance, and technical aspects of cell culture. In addition to this expected biological variability, batch-to-batch variation in key commercial reagents (e.g. Matrigel, CHIR99021, FGF9, and E6/E8 media and supplements) can lead to more pronounced effects, including impaired maintenance of pluripotency or failure of nephron formation. While these outcomes are widely recognised and routinely managed in the field, they are not always explicitly described. To ensure consistency of baseline tissue composition prior to assessing injury responses, differentiations were screened using predefined morphological criteria to determine inclusion or exclusion of entire differentiation batches prior to downstream analyses. Inclusion was based on objective features, including the presence of epithelial domains on day 7 of differentiation, appropriate growth and absence of gross morphological abnormalities in established organoids (d12-

d18). Representative images of included and excluded differentiations are shown in Supplementary Fig. 1A.

Macrophage differentiation and integration with kidney organoids

iPSC-derived macrophages were differentiated from the MCRIi0001-A (PB001.1) line. MCRIi0001-A iPSCs were maintained in Essential 8 medium (Thermo Fisher, cat. no. A1517001) on Matrigel-coated dishes and passaged at 70–80% confluence. Macrophages were differentiated from Myeloid progenitors arising from embryoid body (EB) differentiations based on established protocols (4-7) and following the approach reported by Joshi et al., (4). Briefly, iPS cells were dissociated at Day 0 with 0.5 mM EDTA, collected by centrifugation (200 g, 4 min), and 1–1.5 × 10^6 cells were replated for embryoid body (EB) formation in non-coated 10 cm dishes in E8 medium on an orbital shaker. Directed differentiation of mesoderm, hemogenic endothelium and monocyte progenitors was achieved by supplementing E8 media with combinations of growth factors and cytokines as follows: **Day 0-1**: 20ng/mL BMP4 (R&D Systems, cat. no. 314-BP), 40ng/mL VEGFA (Thermo Fisher, cat. no. 100-20), 40ng/mL SCF (Thermo Fisher, cat. no. 300-07), 10ng/mL FGF2 (Thermo Fisher, cat. no. 100-18B), 5ng/mL Activin A (R&D Systems, cat. no. 11348-AC) and 0.2µM CHIR99021 (Tocris Biosciences cat. no. 4423). **Day 1-2**: 20ng/mL BMP4, 40ng/mL VEGFA, 40ng/mL SCF, 10ng/mL FGF2, 0.2µM CHIR. **Day 3-8**: 20ng/mL BMP4, 40ng/mL VEGFA, 40ng/mL SCF, 10ng/mL FGF2. **Day 8-10**: 20ng/mL VEGFA, 20ng/mL SCF, 10ng/mL FGF2, 25ng/mL IL3 (Thermo Fisher, cat.no. 200-03). **Day 11+**: 20ng/mL VEGFA, 20ng/mL SCF, 10ng/mL FGF2, 25ng/mL IL3, 50ng/mL FLT3L (Thermo Fisher, cat.no. 300-19) 25ng/mL GM-CSF (Thermo Fisher, cat.no. 300-03). Monocyte progenitor cells arising from EB cultures were confirmed by flow cytometry for CD14 from day 11. Progenitor cells were isolated from the supernatant of EB cultures and plated at 1 × 10^5 cells/well in a 6-well plate in RPMI + 10% FBS + 100 ng/ml M-CSF-1 (Thermo Fisher, cat. No. 300-25) for macrophage differentiation and maturation. Macrophages were matured after 4 days, with medium changed every 3 days for extended culture. Successful differentiation was defined by a CD45+/CD14+/CD68+ phenotype (>95% purity), assessed via flow cytometry (BioLegend, cat. nos. CD14 301830, CD45 368512, CD68 333816). Prior characterization from ourselves and others demonstrates iPSC differentiated in this manner resemble yolk-sac derived (tissue resident) macrophages (6). Being derived in vitro, the cells are not exposed to donor or environmental stimuli that may otherwise lead to batch variation polarization.

Integration of macrophages was performed on day 7 of kidney organoid differentiation. After single cell dissociation with TrypLE Select Enzyme (1X) (Thermo Fisher Scientific), 7500 macrophages and 142500 cells from a Day 7 kidney differentiation were combined in microtubes, mixed and

centrifuged to form organoids containing 5% macrophages: 95% kidney progenitors (at day 7). After centrifugation, organoids were transferred to a transwell membrane for culture as previously described.

Hypoxic Injury

iPSCs and organoids were cultured in standard conditions in a humidified incubator (37C, 5% CO_2, 19% O_2, 76% N_2) until day 18 of differentiation. Control organoids were maintained at 19% O_2 from this point, while experimental groups were cultured in a humidified incubator under hypoxic incubators (37C, 5% CO_2, 1% O_2, 94% N_2) for 48h then harvested immediately (d20) or after a 5-day recovery at 19% O_2 (d25). For bulk RNA-sequencing, quantitative PCR, proteomics and metabolomics studies, organoids were snap-frozen, for immunostaining and spatial transcriptomics, organoids were fixed in 4% paraformaldehyde (PFA), at d20 and d25.

Quantitative PCR

RNA isolation and purification was performed with the Isolate II RNA Mini Kit (Bioline, cat no. BIO-52072), following the manufacturer's instructions. RNA quantification was carried out using NanoDrop ND-1000 (ThermoFisher). cDNA was synthetized using the QuantiTect Rev. Transcription kit (QIAGEN, cat no. 205311). Quantitative PCR was performed using the Qiagen Quantinova SYBR green (Qiagen, cat no. 208054) on a 384-well Biorad unit. Gene expression was calculated using ΔCT method and beta-actin (*ACTB)* for normalization. Error bars represent standard deviation (SD) of biological replicates of the three cell lines. Data is expressed as mean ± SD and analyzed by analysis of variance (ANOVA) with Tukey's multiple comparisons test. Differences were considered significant at *$P < 0.05$, **$P < 0.01$, ***$P < 0.001$.

Primers used were: HIF1A (F: CATAAAGTCTGCAACATGGAAGGT, R: TTGATGGGTGAGGAATGGGTT), VEGFA (F: TGCAGATTATGCGGATCAAACC, R: TGCATTCACATTTGTTGTGCTGTAG), HAVCR1 (KIM-1) (F: CTGCAGGGAGCAATAAGGAG, R: TCCAAAGGCCATCTGAAGAC), HK1 (F: GAGTTTGACCTGGATGTGGTTGC, R: CCTCCATGTAGCAGGCATTGCT), TGFB1 (F: TACCTGAACCCGTGTTGCTCTC, R: GTTGCTGAGGTATCGCCAGGAA), COL1A1 (F: TCTGCGACAACGGCAAGGTG, R: GACGCCGGTGGTTTCTTGGT), FN1 (F: ACAACACCGAGGTGACTGAGAC, R: GGACACAACGATGCTTCCTGAG), CCL2 (F: AGGTGACTGGGGCATTGAT, R: GCCTCCAGCATGAAAGTCTC), CDKN1A (F: AGGGGACAGCAGAGGAAG, R: GCGTTTGGAGTGGTAGAAATCTG), NANOG (F: AATACCTCAGCCTCCAGCAGATG, R: TGCGTCACACCATTGCTATTCTTC), TBX6 (F:

CATCCACGAGAATTGTACCCG, R: AGCAATCCAGTTTAGGGGTGT), T (F: AGGTACCCAACCCTGAGGA, R: GCAGGTGAGTTGTCAGAATAGGT), SIX2 (F: CTGGAGAGCCACCAGTTCTC, R: GCTGCGACTCTTTTCCTTGA), OSR1 (F: CCTTCCTTCAGGCAGTGAAC, R: CGGCACTTTGGAGAAAGAAG), EYA1 (F: ATCTAACCAGCCCGCATAGC, R: GTGCCATTGGGAGTCATGGA), LHX1 (F: ATGCAACCTGACCGAGAAGT, R: CAGGTCGCTAGGGGAGATG), GATA3 (F: GCCCCTCATTAAGCCCAAG, R: TTGTGGTGGTCTGACAGTTCG), SLC12A1 (F: AGTGCCCAGTAATACCAATCGC, R: GCCTAAAGCTGATTCTGAGTCTT), UMOD (F: TGACCAACTGCTATGCCACACC, R: GAACATCTGGACGGAAAATCGGC), LRP2 (F: AAATTGAGCACAGCACCTTTGA, R: TCTGCTTTCCTGACTCGAATAATG), CALB1 (F: TCCAGGGAATCAAAATGTGTGG, R: GCACAGATCCTTCAGTAAAGCA), NPHS1 (F: AGTGTGGCTAAGGGATTACCC, R: TCACCGTGAATGTTCTGTTCC), PECAM1 (F: GCAGCATCGTGGTCAACATAA, R: GCAGGACAGGTTCAGTCTTTCA), GDF15 (F: GTTAGCCAAAGACTGCCACTG, R: CCTTGAGCCCATTCCACA), PPARGC1A (F: GTCACCACCCAAATCCTTAT, R: ATCTACTGCCTGGAGACCTT), ACTB (F: GTTACAGGAAGTCCCTTGCCATCC, R: CACCTCCCCTGTGTGGACTTGGG).

Western blot

The nuclear fraction of pools of 3 organoids subjected to 24 h and 48 h of hypoxia was used for Western blot analysis. Organoids were sonicated and lysed in 0.4 ml of RIPA lysis buffer. Samples were centrifuged at 875 g at 4°C for 30 min to remove cell debris. Protein concentrations were measured using a modified Lowry protein assay with BSA as the protein standard (DC Protein Assay Kit, Bio-Rad). Extracted proteins were separated by electrophoresis on a 10% SDS–PAGE gel and then transferred to a PVDF membrane. After blocking for 30 min at 4°C in blocking buffer (5% skim milk powder in PBS with 0.1% Tween-20), the membrane was incubated overnight with a mouse monoclonal anti-HIF-1α primary antibody (1:300, Novus Biologicals, cat. No. NB100-105). The membrane was then washed and incubated for 30 min at room temperature with a goat anti-mouse HRP secondary antibody (1:5000, Novus Biologicals, cat. No. HAF007). β-actin was used as a loading control (1:5000, Novus Biologicals, NB600). Immunoreactive bands were detected using the ChemiDoc MP Imager (Bio-Rad).

Bulk RNA-seq and Data Analysis

Total RNA was isolated from kidney organoids on d20 and d25 of differentiation in triplicates using the Isolate II RNA Mini Kit (Bioline, cat no. BIO-52072). Sequencing was performed at MHTP Medical Genomics at Monash University using the 3'-Multiplex RNA-seq method, an in-house

developed method that captures and sequences the 3'end of pA transcripts, suitable for multiplexing. RNA integrity was assessed with Bioanalyzer (Agilet Technologies) (see Supplemental Table 7 for RIN scores). All samples were processed starting with 50 ng of total RNA. Libraries were made by individual first strand synthesis to add the i7 index sequences indicated in Supplemental Table 3. Library construction of pooled cDNA (tagged with i7 indexes) was completed by adding the full P7 and P5 arms by tagmentation and PCR. Quality control (QC) for the library pool included analysis concentration and quality using a Qubit, Bioanalyzer and qPCR. It had a mean size of 466bp at a concentration of 7.1 nM. The library pool was loaded at 1000pM (based upon size adjusted qPCR) for on-board denaturation and clustering which resulted in a loading metric of 99.7 and 90% >Q30. 463.2 Million reads were assigned to indexes. Numbers of reads assigned to each sample are shown in Supplemental Table 3**.** Libraries were run on Illumina NextSeq 2000.

Sequencing data were indexed to the human GRCh38 reference genome using the STAR alignment tool v.2.7.10 and human Ensembl GTF annotation file v.106 (8). Subsequently, fastq files were aligned to the indexed genome to generate BAM files. The BAM files were then sorted and indexed using "samtools" software v.1.15.1 (9). To eliminate duplicated reads based on Unique Molecular Identifiers (UMIs), the "umi_tools" software v.1.1.2 was employed (10). Count data was generated using "featureCounts" tool v.2.0.1 on sorted deduplicated BAM files (11). The resulting count data were imported into the R statistical programming environment to assemble the gene expression matrix. "biomaRt" R package v.2.46.3 was used to map Ensembl gene Ids to gene symbols (12). Data were normalized using the CPM method and differentially expressed genes were recognized using voom estimated weights and limma R package (13, 14) and Degust software (15) (BH adj-p-value < 0.05 & absolute LogFC > 0.5).

Differentially expressed genes were used to perform enrichment analysis using the DAVID online tool (16). Further analysis was performed with clusterProfiler R package v.4.6.1, taking ranked symbols based on LogFCs. For GSEA analysis, normalized gene expression profiles were imported into GSEA tool v.4.2.3 (17) employing the MSigDB database (18).

Single cell RNA-seq and Data Analysis

12 samples from iPSC-derived kidney organoids were labelled with Cell Plex (10x Genomics) Cell Modified Oligonucleotides (CMO) for multiplexing: n1_d20 (CMO301), n2_d20 (CMO302), n3_d20 (CMO303), h1_d20 (CMO304), h2_d20 (CMO305), h3_d20 (CMO306), n1_d25 (CMO307), n2_d25 (CMO308), n3_d25 (CMO309), h1_d25 (CMO310), h2_d25 (CMO311), and h3_d25 (CMO312). These 12 samples correspond to 4 conditions with 3 replicates per condition and a pool

of 3 organoids for each replicate. Libraries were prepared using 10X Genomics Chromium Single Cell 5' V3.1 chemistry, for GEX and FB/CellPlex libraries (10X Genomics; San Francisco CA, USA), according to the manufacturer's instructions and sequenced on MGITech MGISEQ2000RS hardware using MGIEasy V3 chemistry for 100 bp paired-end sequencing at the Monash Micromon Genomics platform.

Raw Sequencing data was processed, and samples were demultiplexed using CellRanger multi (v.7.0) (19), with the CMOs provided (Supplemental Table 8). Reads were aligned to the Homo sapiens GRCh38 (2020-A) reference. Feature counts per cell, cell barcodes and mapping rate were used to evaluate cell quality. An average of 975 cells were captured per sample with high mapping rates to reference (>98%) and an average of 8,251 features per cell (Supplemental File 3). Quality control measures, preprocessing steps, and all subsequent analysis and related code is documented and available in a public github repository, which also features an interactive marker lookup site to interrogate gene expression within experimental groups and cell types https://github.com/MonashBioinformaticsPlatform/sc-hyp-org.

Further investigation of data quality and analysis was performed using R package Seurat (v. 4.3.0) (20). The distribution of the number of features per cell (unique UMIs per cell), number of reads per feature (UMI counts), and mitochondrial content was assessed to determine the quality of the cells. A threshold of cells with more than 200 features (unique UMIs per cell) was applied. Mitochondrial gene expression in kidney cells is higher than other organs (up to 50%), and was expected to be increased in our experiment. Therefore, mitochondrial expression was not considered as sign of dying cells but a response of kidney cells to the conditions in this experiment and no mitochondrial percentage threshold was used to filter out cells. Normalization of reads per cells (UMI counts) was performed using the SCT transform method (21). An elbow plot of a Principal Component Analysis (PCA) was used to visualize dimensionality; 15 dimensions represented most of the variability. Dimensionality reduction was performed using Uniform Manifold Approximation and Projection (UMAP) with 15 dimensions.

The d20h group was profoundly affected by hypoxic exposure and formed unique clusters, while data from the d20n, d25n/n and d25 h/n groups clustered together. Various strategies were attempted to integrate the d20h group and enable comparison of cell-type specific changes in comparison to control data within the same cell type-cluster. A cluster tree (22) was generated to evaluate the clustering resolutions from 0 to 2. A resolution of 0.7 was selected as this resolution captured most of the clusters' variability without diverging into smaller subclusters with low number of cells (<18 cells)

(Supplemental Figure 9A). Second, 30 to 50 of the most variable gene markers per cluster in the normoxic clustering were used as anchor genes to identify clusters in hypoxic groups. Finally, the cluster labelling was informed and refined by observing the segregation of the clusters, identification of gene markers in literature, comparison to the Enrichr database (23) and the use of the R package DevKidCC (24). Gene set enrichment analysis was used to identify clusters defined by signatures of proliferation and cellular stress, as well as known 'off-target' neural and myocyte-like populations (23). Changes in the proportion of cell populations was assessed using the propeller method from the R package speckle (25). Differentially Expressed Genes (DEGs) were identified using the pseudobulk approach (26). The pseudobulk analysis was performed using the R packages limma (27) and edgeR (28). scRNAseq Sequencing data was submitted to GEO Dataset under the ID GSE236379.

Targeted analysis of proximal tubule cell states

Data loading and preprocessing

The integrated Seurat object was loaded and updated to Seurat v4.4.0. For all downstream analyses, the RNA assay was set as default and log-normalised using NormalizeData() (method = "LogNormalize", scale factor = 10,000) prior to module scoring and differential expression analysis, as required by AddModuleScore().

PT cell redefinition

PT identity was assessed by module scoring using Seurat's AddModuleScore() function applied to a panel of 20 canonical PT marker genes: *LRP2, GATM, PDZK1, GPX3, FUT9, SLC27A2, SPP1, SLC5A8, AMN, SLC17A1, SLC3A1, SLC4A4, SLC39A5, CUBN, TINAG, FMO1, SMIM24, SLC6A13, APOM, HNF4G*. All 20 genes were detected in the dataset. Scores were computed across all cells using a random seed of 42. Cells were classified as PT if their module score exceeded mean + 1 SD (threshold = 0.4715; calculated across all cells) and originated from one of three biologically plausible clusters: 12_Proximal_Tubule, 5_Distal_Tubule, or 10_Early_Nephron. This combined filter identified 271 redefined PT cells compared to 163 in the original annotation. Cell-level reclassification data, including original cluster labels, PT module scores, and final assignments, are provided in Supplementary File 3. To check the PT module score–based reclassification, an independent 28-gene PT marker set, derived from the original PT cluster marker list, was evaluated across original and reclassified PT and DT clusters at day 25. This independently confirmed elevated PT marker expression in the injured DT cluster prior to reclassification and its reduction following reclassification. Validation set: *GK, GIPC2, HGD, ENTPD5, CDKN1A, TMEM37, TMEM150A, CHPT1, SLC16A4, LGMN, AMACR, SLC37A4, TMEM176A, DAB2, SLC1A1, FGGY, ENPEP, PECR, SLC9A3R1, TMEM176B, CISD3, FUOM, SCLY, PRLR, ACY1, SLC44A4, HNF1B, TCEA3*.

Differential expression analysis

Differential expression between d25n (normal_d25; n = 43 redefined PT cells, 3 replicates) and d25h/n (hypoxic_d25; n = 115 redefined PT cells, 3 replicates) was performed using a pseudobulk approach. Raw RNA counts were aggregated at the level of biological replicates using Seurat's AggregateExpression() function. Genes expressed in fewer than 2 samples were excluded. Differential expression analysis was performed using DESeq2 v1.42 with a design formula of ~treatment, with normal_d25 as the reference level. Results were extracted using a contrast of hypoxic_d25 versus normal_d25 with an alpha threshold of 0.05. Log2 fold changes and Benjamini–Hochberg adjusted p values are reported. Full DEG results are provided in Supplementary File 3.

Gene set scoring

Module scores for custom and published gene sets were calculated using AddModuleScore() (seed = 42) for the following gene sets: Custom PT markers (20 genes, as above); Custom injury markers: SOX9, CDKN1A, VCAM1, SPP1, PROM1, ICAM1; KPMP healthy PT signature (285 genes; Lake et al. 2023, Supplementary Table 11, PT cluster); KPMP adaptive PT signature (447 genes; Lake et al. 2023, Supplementary Table 11, aPT cluster). Gene set overlap with the dataset was 20/20, 6/6, 284/285, and 445/447, respectively. Statistical comparisons between conditions were performed using the Wilcoxon rank-sum test with Benjamini–Hochberg correction implemented in the rstatix package v0.7.2.

Subpopulation analysis

Redefined d25h/n PT cells were subsetted and reprocessed independently. Normalisation, variable feature selection (nfeatures = 2,000), scaling, PCA (npcs = 20), and UMAP (dims = 1:15) were performed using standard Seurat workflows. A shared nearest neighbour graph (dims = 1:15) was constructed, and Louvain clustering at resolution 0.8 identified two subpopulations (cluster 0, cluster 1). Cluster marker genes were identified using FindAllMarkers() with a minimum expression fraction of 0.25 and log2 fold-change threshold of 0.25, retaining only positive markers. Full marker gene lists are provided in Supplementary File 3.

Conservative proximal tubule (PT) reclassification using UCell-based module scoring

All data presented in the main manuscript and supplementary data derives from the initial PT reclassification strategy detailed above. To independently validate this, a second, approach was implemented using more conservative thresholds and UCell-based module scoring

(AddModuleScore_UCell, UCell package), which ranks gene expression within individual cells and anchors classification to a biologically defined reference population.

Canonical PT marker genes were partitioned into functionally distinct modules (canonical identity, transporters, metabolic, regulatory, specialised, and injury-associated) to enable independent evaluation of PT identity features. This partitioning was necessary as some modules (e.g. regulatory or injury-associated genes) substantially influenced composite scores. Evaluating modules separately allowed canonical identity to be used for classification, while transporter and metabolic signatures provided independent validation of functional PT characteristics. PT assignment was defined relative to normoxic PT cells, using the 25th percentile of canonical PT UCell scores within the "12_Proximal_Tubule" clusters as the threshold. This criterion requires that reclassified cells retain canonical PT marker expression exceeding the lower quartile of uninjured PT cells, anchoring classification in preserved epithelial identity. As a result, this approach is inherently conservative, excluding a substantial proportion of normoxic PT cells and an even greater fraction under injury conditions, and thus likely under-representing injured PT populations with reduced marker expression. Consistent with this reduced sensitivity, fewer cells were reassigned as PT compared to the initial mean + 1 SD thresholding method in the d25h/n condition. Nevertheless, residual PT-like cells were still identified within clusters annotated as distal tubule following injury, supporting the presence of cells with attenuated but persistent PT identity.

Comparison of module scores across reclassified populations indicated that canonical PT scores in reassigned cells were intermediate relative to the original d25h/n PT cluster, and that transporter and metabolic module scores were reduced, consistent with impaired functional identity. Together, these data support a model in which injury induces divergent epithelial states, with a subset of cells partially restoring PT identity, while others retain reduced differentiation and metabolic capacity. Analysis code and results are provided in the linked GitHub repository.

Taken together, the initial mean + 1 SD–based PT module scoring approach is likely permissive, whereas the UCell-based, reference-anchored strategy is comparatively stringent. Concordance between approaches in identifying additional PT-like cells with reduced canonical identity and metabolic function supports the robustness of the observation that PT identity is variably preserved following injury, while differences in the number of reclassified cells highlight the sensitivity of classification to thresholding in datasets with limited epithelial representation.

Preparation of Samples for Untargeted Metabolomic and Proteomic Analysis

Proteomic and metabolomic profiling were performed on matched organoid samples, with metabolites and proteins extracted from the same organoids and analysed separately. Eight organoids per group were processed for extraction; all eight were used for metabolomic analysis to account for higher variability in this method, while a subset of four per group was used for proteomic analysis. All organoids were derived from a single differentiation. Without disrupting the organoid architecture, individual organoids were transferred to a low binding 1.5 mL Eppendorf tube. Organoids were washed with 1 mL of ice cold 0.9 % NaCl. After a 10 min centrifugation at 1000 x g, 4 °C, all the saline solution was removed and organoids frozen in liquid nitrogen. Frozen pellets were resuspended in 200 µL of extraction solvent consisting of 2:6:1 chloroform:methanol:water with a mixture of internal standards (CHAPS, CAPS, PIPES and TRIS; 2 µM each). Samples were frozen in liquid nitrogen and thawed on ice three times then agitated on a vibrating mixer for 20 minutes at 4°C before centrifugation at 20,000 × g and 4°C for 10 min. The supernatant was transferred to a glass vial for LC-MS based metabolomic analysis, while the pellet of four samples was used for proteomics analysis as described below.

<u>Cell Lysis and Enzymatic Digestion</u>

Protein pellets were resuspended in 1% (w/v) Sodium deoxycholate (SDC) (Sigma, D6750-100G), 100mM Tris pH 8.1 buffer, boiled at 95°C for 5 min. Subsequently, DNA was sheared using probe ultra-sonication at 50% amplitude (Q125 sonicator, Q-Sonica, Newtown, Connecticut, USA) with three rounds of 10-second intervals. Protein concentration was measured using a BCA kit according to the kit's manual (Pierce BCA Protein Assay Kit #2322). TCEP (500 mM Bond-Breaker Thermo, #77720) to a final concentration of 10 mM was added to denature samples and incubated for 30 min at 50°C. Then samples were alkylated by adding chloroacetamide to a concentration of 40 mM and incubate for 20 min at RT in the dark. Finally, 1:100 of trypsin (Promega, V5111) was added and incubated at 37°C with shaking overnight. Trypsin digestion was stopped by adding formic acid (Sigma, 56302-50ML-F) to a final concentration of 1%, and pH was ensured to be pH ~3. Samples were centrifuged at 5 min with max speed and supernatants were transferred to new tubes.

<u>Stage tip SDB Clean-up Protocol</u>

Stage-Tipping was used to clean the peptide digest using SDB-RPS (Empore) (29). Samples were reconstituted in 20 μL of loading buffer (2% acetonitrile, 0.1% TFA and 97.9% MilliQ water) (Thermo Fisher Scientific, Optima LC/MS, A955-4) containing iRT peptides, sonicated for 10 minutes in a sonicator water bath, centrifuged at max speed for 5 minutes and transferred into MS vials to be loaded into the mass spectrometer.

Liquid Chromatography Mass Spectrometry for Proteomic Analysis

Using a Dionex UltiMate 3000 RSLCnano system equipped with a Dionex UltiMate 3000 RS autosampler, an Acclaim PepMap RSLC analytical column (75 µm x 50 cm, nanoViper, C18, 2 µm, 100Å; Thermo Scientific) and an Acclaim PepMap 100 trap column (100 µm x 2 cm, nanoViper, C18, 5 µm, 100Å; Thermo Scientific), the tryptic peptides were separated by increasing concentrations of 80% acetonitrile (ACN) / 0.1% formic acid at a flow of 250 nl/min for 158 min and analyzed with an Orbitrap Fusion Tribrid mass spectrometer (ThermoFisher Scientific). The instrument was operated in data dependent acquisition mode to automatically switch between full scan MS and MS/MS acquisition. Each survey full scan (m/z 375–1800) was acquired in the Orbitrap with a resolution of 120,000 (at m/z 200) after accumulating ions with a normalized AGC (automatic gain control) target of 250% and an automated maximum injection time. The cycle time was set to two seconds with the most intense multiply charged ions (z = 2-7) sequentially isolated and fragmented in the collision cell by higher-energy collisional dissociation (HCD) with a resolution of 30,000, an AGC target of 400% and an automated maximum injection time. Dynamic exclusion was set to 15 seconds.

Proteomic Mass Spectrometric Data Analysis

The mass spectrometric raw data files were analyzed with the Fragpipe software suite 17.1 (MSFragger version 3.4, Philosopher version 4.1.1 (30, 31) to obtain protein identifications and their respective label-free quantification (LFQ) values. The standard label free quantification match-between-runs (LFQ-MBR) workflow was applied with no changes to workflow, employing IonQuant (Version 1.7.17) (32) and the MaxLFQ method of protein abundance calculations (29). Searches were performed against the Human SwissProt proteome (accessed June 2020) and with common contaminants. The proteomics data were further analyzed using LFQ-Analyst which performed data manipulation and statistical tests with standard parameters (see (33)).

Liquid Chromatography Mass Spectrometry for Metabolomic Analysis

Untargeted metabolomic analysis was performed with a Dionex RSLC3000 UHPLC coupled to a Q-Exactive Plus MS (Thermo Fisher Scientific). The chromatography utilized a ZIC-p(HILIC) column 5µm, 150 x 4.6 mm (Merck Millipore, Australia) at 25 °C. A gradient elution of 20 mM ammonium carbonate (A) and acetonitrile (B) (linear gradient time-%B: 0 min-80%, 15 min-50%, 18 min-5%, 21 min-5%, 24 min-80%, 32 min-80%) was utilized. Flow rate was maintained at 300 µL/min. Samples were kept in the autosampler (6°C) and 10 µL was injected for analysis. MS was performed at 70,000 (at m/z = 200) resolution operating in rapid switching positive (4 kV) and negative (−3.5

kV) mode electrospray ionization (capillary temperature 300°C; sheath gas flow rate 50; auxiliary gas flow rate 20; sweep gas 2; probe temp 120°C). For accurate metabolite identification, a standard library of ~500 metabolites were analysed before sample testing and accurate retention time for each standard was recorded. This standard library also forms the basis of a retention time prediction model used to provide putative identification of metabolites not contained within the standard library (34). Acquired LC-MS data was processed in an untargeted fashion using open source software IDEOM, which initially used msConvert (ProteoWizard)(35) to convert raw LC-MS files to mzXML format and XCMS to pick peaks to convert to .peakML files (36). Mzmatch was subsequently used for sample alignment and filtering (37). On importing the aligned peak data into IDEOM, the intensity was normalised to the median peak intensity before running the identification macro to provide identification of the chromatographic features (36).

Organoid Tissue Processing and Immunostaining

Organoids were washed with DPBS (no calcium, no magnesium, Thermo Fisher Scientific). Forcibly pipetting DPBS around the organoids caused the organoids to detach from the transwell without causing tissue damage. Organoids were then placed in a low attachment 48-well plate (Thermo Fisher Scientific) with a cut P1000 tip. For whole mount staining, organoids were fixed in 2 % (paraformaldehyde) PFA for 20 min and blocked for 30 min (5 % Donkey serum, 0.1 % Triton X-100, made up in PBS) at room temperature. Organoids were then placed overnight with the primary antibody or staining reagent at 4 °C in blocking solution. Kidney organoids were stained with LTL-biotin (Vector Laboratories B-1325, dilution 1:500), Rabbit anti-GATA3 (Cell Sig – 5852, dilution 1:300), Sheep anti-UMOD (Bio-rad - 8595-0054, dilution 1:300), Rabbit anti-MAFB (Sigma HPA005653, dilution 1:300), Sheep anti-NPHS1 (R&D - AF4269-SP, dilution 1:300), Rabbit anti-PBX1 (Cell Sig - 4342, dilution 1:300), Mouse anti-MEIS1 (Active Motif - ATM39795, dilution 1:300), Rabbit anti-PECAM1 (Sigma - SAB4502167, dilution 1:300), Goat anti-KIM1 (R&D - AF1750, dilution 1:100), Mouse anti-CK (cytokeratin, Abcam - ab115959, dilution 1:300), Rabbit anti-ICAM1 (Cell Sig – 4915, dilution 1:300), Goat anti-Collagen IV (Bio-Rad - 1340-01, dilution 1:400), Rabbit anti-VCAM1 (Abcam - ab134047, dilution 1:600), Sheep anti-SOX9 (38) (dilution 1:600, gift from Dagmar Wilhelm), Mouse anti-EPCAM-488 (Biolegend – 324210, dilution 1:300), Rabbit anti-CDKN1A (Cell Signalling - 2947T, dilution 1:300). Primary antibodies were washed off with PBS 3 x 15 min. Organoids were then incubated overnight at 4 °C with the secondary antibody (all 1:1000 dilution in blocking solution): donkey anti-Rabbit IgG (Thermo Fisher Scientific, A1004200); donkey anti-Sheep IgG (Thermo Fisher Scientific, A21448); Alexa Fluor 568 streptavidin (Thermo Fisher Scientific, S11226); donkey anti-Mouse IgG (Thermo Fisher Scientific, A32787, A32766); donkey anti-Goat (Thermo Fisher Scientific, A32849). DAPI or Hoechst were

included with secondary antibodies to stain nuclei (1:1000 dilution). Fluorescence images were captured using a 3i CSU-W1 T2 Yokogawa Spinning disk confocal microscope or a VS200 slide scanner (Olympus). Images were pseudo-coloured to permit overlay, cropped, sized in ImageJ (NIH), QuPath, or Imaris, with final cropping in Adobe Illustrator.

For paraffin embedded tissue, organoids were fixed in 2 % PFA at 4 °C overnight. Samples were processed using a Leica Peloris II Tissue Processor at Monash Histology Platform. Briefly, organoids were processed to paraffin wax through 80% Ethanol and multiple stations of 100% Ethanol. Tissues were then dehydrated in several changes of Xylene before being infiltrated through multiple stations of Paraplast paraffin wax and embedded in the same. Immunostaining for collagen type I (Cell Sig – 72026, dilution 1:200) and PAX8 (MA5-32382, dilution 1:400) was performed on 4 µm thick sections using antigen retrieval with 0.1 mol/L sodium citrate, pH 6.0, and a three-layer avidin-biotin peroxidase complex staining method (Vector Laboratories), as described (39). Tubular damage was assessed on organoids using Periodic acid-Schiff (PAS) stain in sections (2 µm thick) of PFA-fixed tissue. Optimal cutting temperature compound (OCT)-embedded frozen sections was used for Oil Red O staining.

Image analysis

Imaging parameters and display settings were kept constant across all samples within each experiment. Images were imported into Imaris, and surface renderings were generated to define tubular structures (Cytokeratin, LTL, or EPCAM) or podocyte structures (MAFB and NPHS1). For analysis of ICAM1 and KIM1 (Imaris v9.4), tubular structures were segmented using intensity thresholding based on Cytokeratin or LTL staining. Thresholds were empirically determined and then applied uniformly across all images within each experiment. For analysis of CDKN1A, SOX9, VCAM1, MAFB, and NPHS1, machine learning–assisted segmentation tools (Imaris v10.0.2) were used to define LTL^+ or $EPCAM^+$ tubules, or $NPHS1^+/MAFB^+$ podocyte structures. Segmentation parameters were optimised on representative images and then applied consistently across the full dataset. For nuclear-localised proteins (CDKN1A, SOX9, MAFB), a pseudo-nuclear segmentation approach was used. Tubular segmentations were applied as masks to isolate nuclear staining (DAPI or Hoechst), and the Imaris "Spots" function was used to identify nuclear centres and generate spherical regions of defined size approximating nuclear boundaries. Example tubular and nuclear segmentation images are provided in Supplementary Figure 6A. Fluorescence intensity of the protein of interest was then quantified within these regions. Mean fluorescence intensity values within segmented tubules, podocyte structures, or nuclei were calculated for each image and aggregated at the level of individual organoids. Data were plotted in GraphPad Prism, and statistical differences

between groups were assessed using two-tailed Welch's t-tests on replicate means, where replicates represent individual organoids derived from a single differentiation. Independent differentiations were used for ICAM1/KIM1 analyses and for the remaining markers.

Spatial Transcriptomics

Kidney organoids +/- integrated iMacs were generated and subjected to hypoxic injury as described above. Organoids were embedded in HistoGel (Epredia, cat. no. HG-4000-012) as per the Manufacturer's instructions, then processed for paraffin embedding (Ethanol 1 min x5, Xylene 1 min x3, Paraffin x3 17 mins). 5µm paraffin sections were cut from 2-5 organoids per experimental group and mounted directly onto a Xenium slide. Samples were profiled with the 380-gene Xenium Human Immuno-Oncology panel, using the Xenium Cell Segmentation kit (10X Genomics) at the Walter and Eliza Hall Institute Spatial Omics Platform Laboratory, as per the manufacturer's protocols. Raw counts from the 10x Xenium Onboard Analysis software were imported into R, loaded into a Seurat object, and normalized with SCTransform (v. 4.3.0) (37). Principal Component Analysis was performed on the SCT-normalized data, and the top 100 components were used for UMAP visualization. During quality control, multidimensional scaling (MDS) analysis revealed that one d25h/n sample (32419.d25h−Rep1) clustered with d25n controls, while the remaining d25h/n samples formed a distinct group (Supplementary Figure 9E). Due to this discordant transcriptional profile, 32419.d25h−Rep1 was excluded from downstream differential expression analysis. Cell type identities were assigned to Xenium clusters by integrating our organoid scRNAseq reference and spatial data using RCTD (40) and Seurat's anchor-based transfer, with results cross-validated and refined by manual assessment of clusters and marker gene expression on tissue images. Unsupervised graph-based clustering was performed in Seurat at multiple resolutions; final assignments using ρ = 0.8 clusters for nephron cells and ρ = 0.3 for other cell types. Pseudobulk differential expression (DE) analysis was performed by aggregating UMI counts across cells per replicate. Pseudo-bulk counts were $\log_2$-transformed, quantile-normalized, and precision-weighted with voom (limma) (27, 41). Linear models with empirical Bayes moderation (42) were used to identify DE genes at FDR $\leq$ 0.05 for comparisons at the level of sample, cell lineage. Cluster-based DE was performed on the single cell level with an FDR cutoff for DE $\leq$ 0.2. Targeted DE analysis was performed on twenty-four manually defined Regions of Interest (ROIs) containing injured nephrons +/-iMacs in the d25h/n_iMacs organoid group, with cells selected by centroid location. The same pseudo-bulk limma-voom pipeline (FDR $\leq$ 0.05) was used to compare all cells, non-iMac cells, and nephron cells only between +/-iMac ROIs. ROIs with fewer than three relevant cells were excluded.

Supplementary Figures

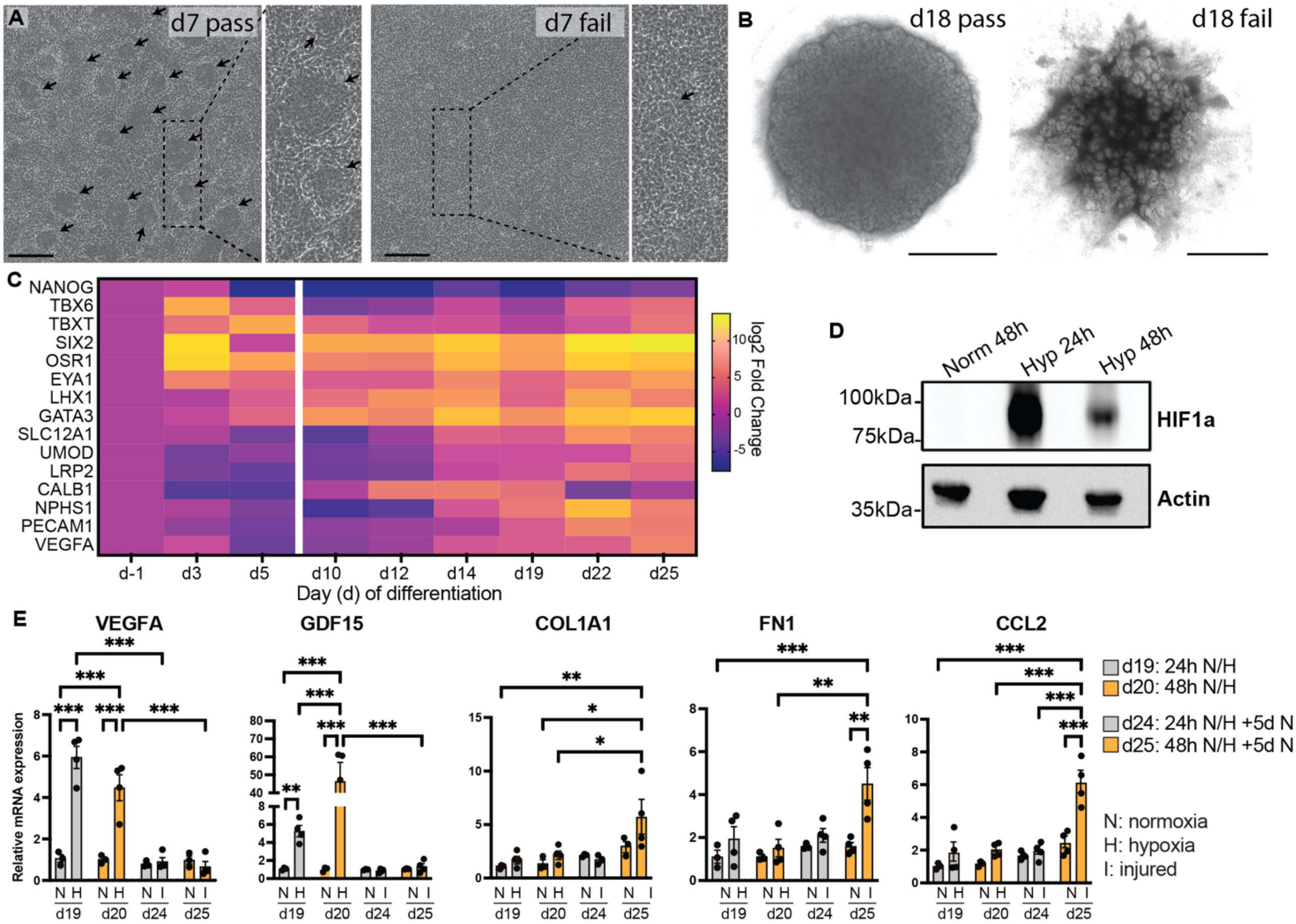

Supplementary Figure 1. Kidney organoid development and hypoxic injury. **A**) Representative bright-field images of day 7 (d7) differentiations meeting inclusion criteria (left; abundant domed/circular epithelial structures, arrows) or failing (right; heterogeneous monolayer lacking defined epithelial domains). Scale bars, 300µm. **B**) Representative day 18 (d18) organoids meeting inclusion criteria (left; uniform morphology with evenly distributed epithelial structures) or excluded (right; irregular morphology with dense regions and reduced epithelial organisation). Scale bars, 1000µm. **C**) Expression of pluripotency, intermediate mesoderm, nephron progenitor and nephron markers from one day before differentiation (day -1) to day 25. Three wells were used as replicates at days 3 and 5; four organoids from days 10–25. Data are log2 fold change normalised to ACTB. **D**) Western blot of HIF1A stabilisation in nuclear extracts following 24 h and 48 h hypoxia versus normoxia. β-actin, loading control. **E**) Representative qPCR data from pilot studies evaluating the duration of hypoxic exposure on markers of the hypoxic response (*VEGFA*), injury (*GDF15*), and persistent injured states (*COL1A1, FN1, CCL2*). Organoids were subjected to 24 h (harvested at d19) or 48 h of hypoxia (harvested at d20), followed by a 5-day recovery period for both groups. Data are mean ± SD (n=3–4), normalised to ACTB and expressed relative to d19n. One-way ANOVA with Tukey's test, except GDF15 (d19 N vs H), assessed by two-tailed Welch's t-test; *p < 0.05, **p < 0.01, ***p < 0.001.

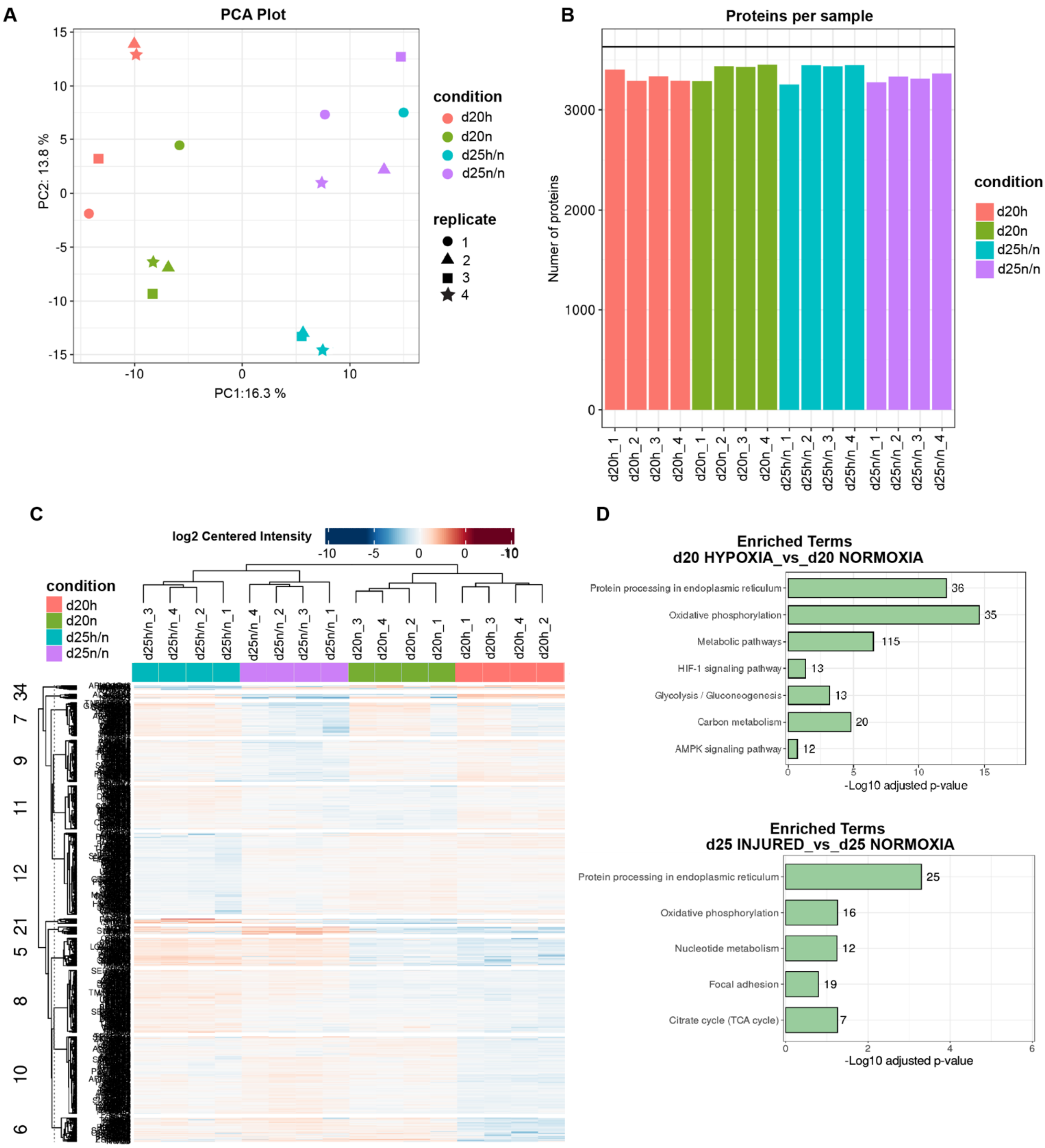

Supplementary Figure 2. Proteomic analysis. A) PCA of the four replicates per condition. **B**) Protein quantification per sample, after pre-processing. **C**) Differential expression analysis in the 12 clusters. Heatmap representing an overview of expression of all significant proteins (rows) in all samples (columns). **D**) KEGG pathway enrichment analysis using the web-based DAVID software (p-value < 0.05) for differentially expressed genes (BH adjusted p-value < 0.05 and absolute LogFC > 0.05). *P*-values were corrected with Benjamini Hochberg approach.

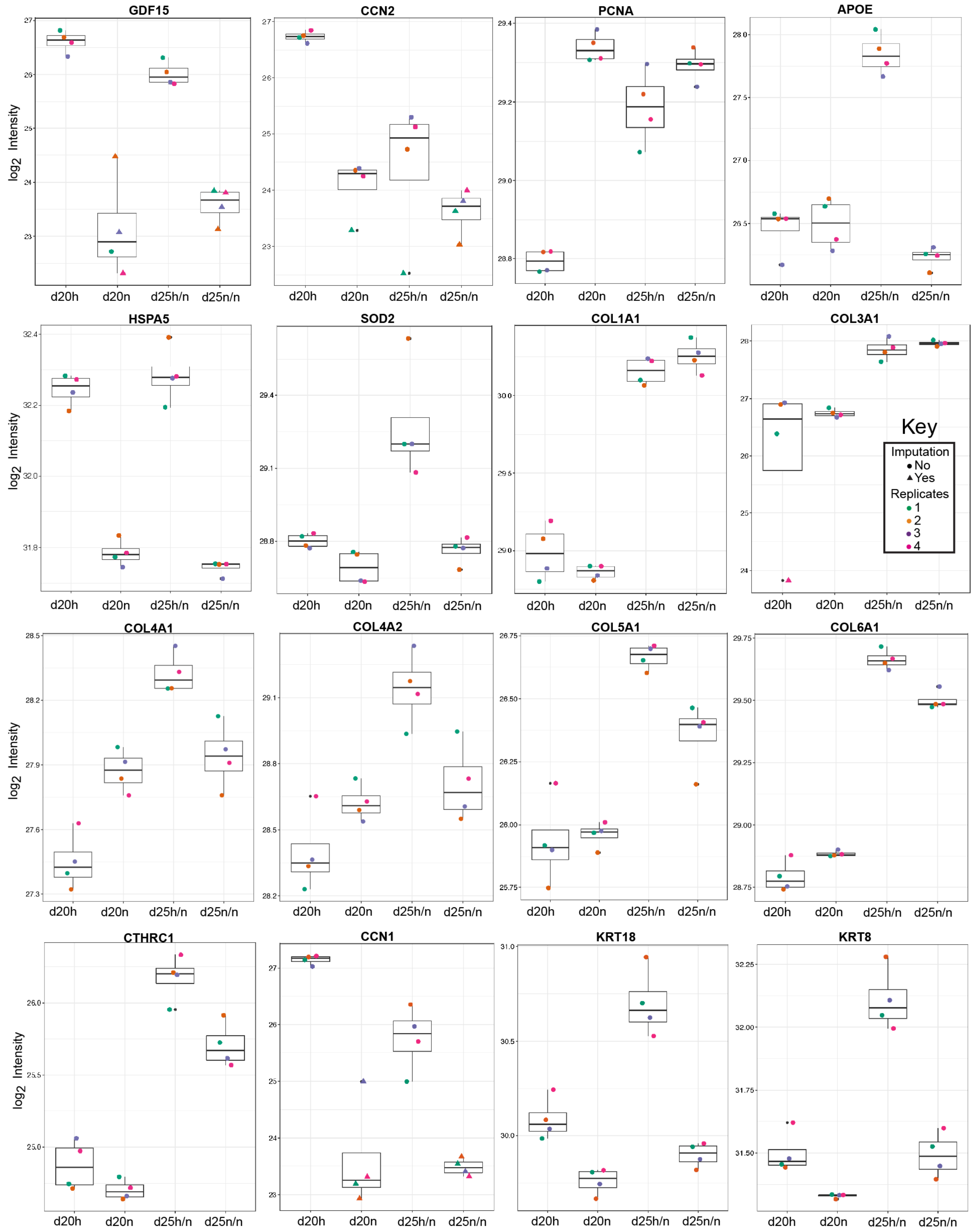

Supplementary Figure 3. Changes in key proteins associated with AKI and persistent injury. Data produced using LFQ-Analyst Proteins. Illustrated proteins associated to kidney injury (GDF15, CCN1, KRT18, KRT8), hypoxia (CCN2, SOD2), cell cycle (PCNA), inflammation (APOE), UPR response (HSPA5), and ECM remodelling (COL1A1, COL3A1, COL4A1, COL4A2, COL5A1, COL6A1, CTHRC1).

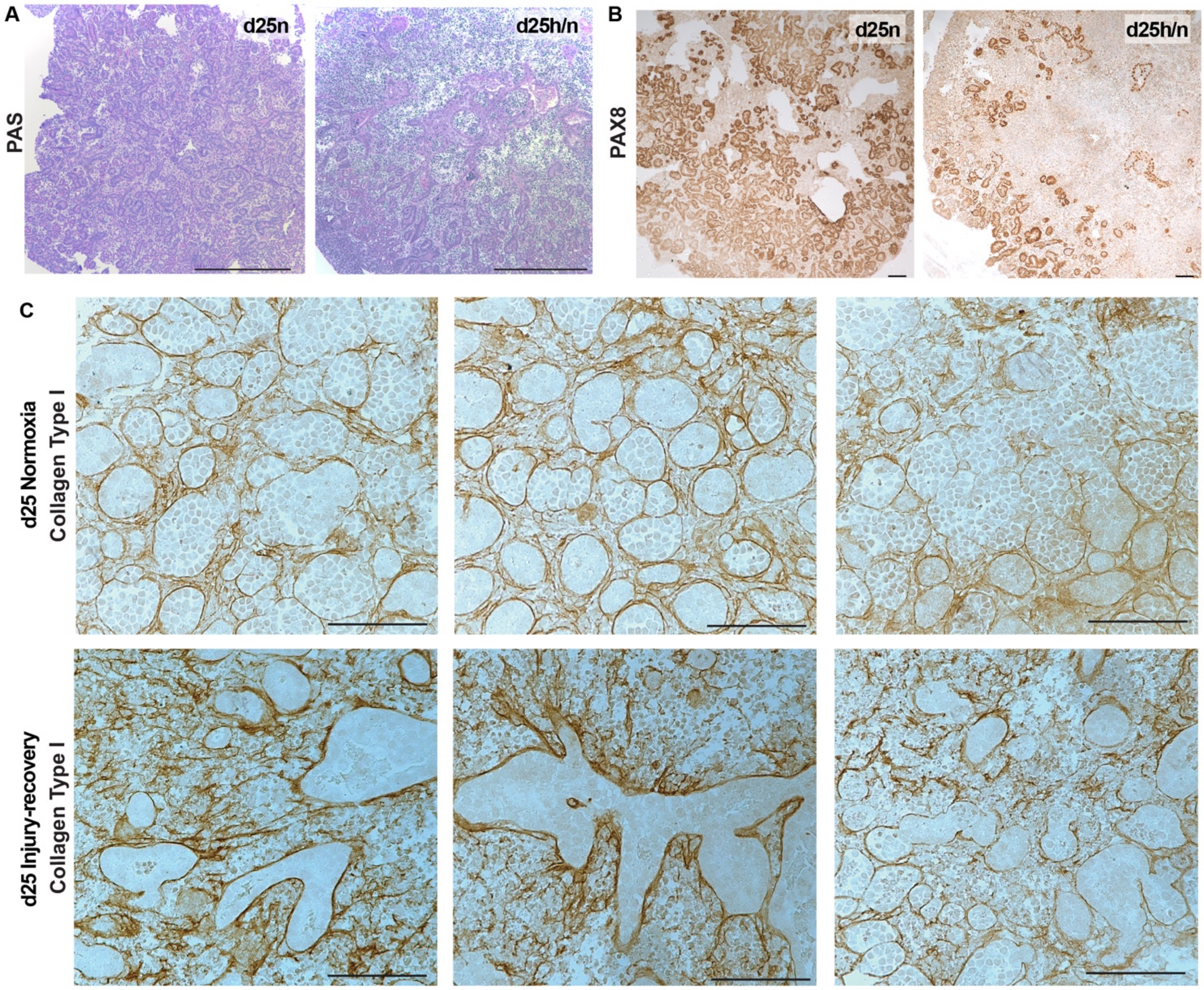

Supplementary Figure 4. Structural disruption and altered extracellular matrix remodelling in injured organoids. A) Representative Periodic acid-Schiff (PAS) staining (n=4 per group) showing reduced tubular structures in d25 injured organoids compared to controls. Scale bars 500 µm. **B**) Representative PAX8 staining (epithelial marker; n=4 per group) showing reduced tubular structures in injured organoids. Scale bars 100 µm. **C**) Collagen type I staining of d25 control and injured organoid sections (n=3, images from independent organoids), showing disrupted tubular morphology and increased interstitial matrix deposition in injured samples. Scale bars 500 µm.

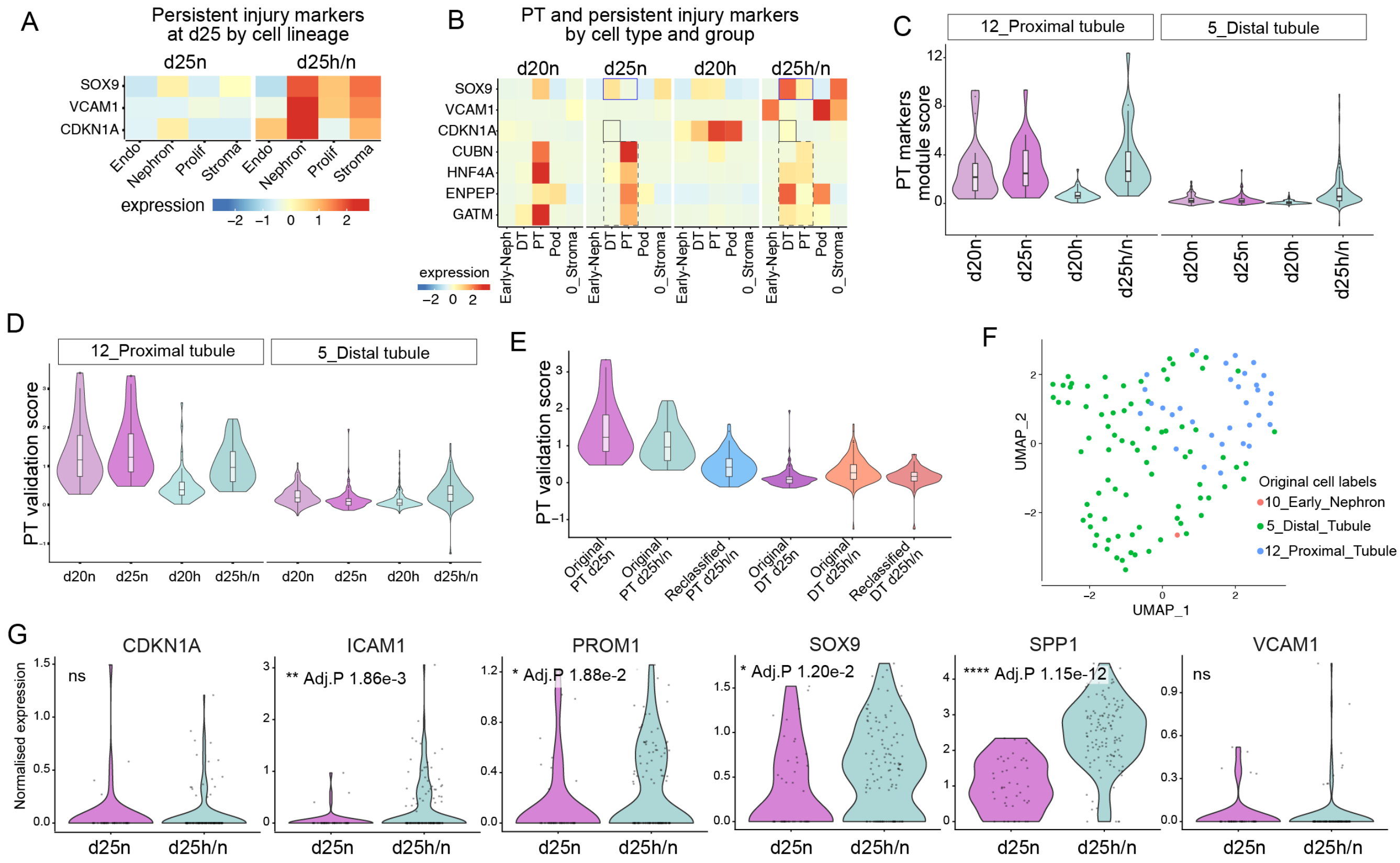

Supplementary Figure 5. PT reclassification, subclustering and injury module scoring. A) Increased expression of persistent injury markers *SOX9, VCAM1, CDKN1A* in d25h/n nephron lineage, compared to proliferating cells, endothelial and stromal lineages. **B)** Proximal tubule (PT) and persistent injury marker expression in nephron clusters and a reference stromal cluster. PT markers are downregulated in d20h, and expression of PT markers is increased in d25h/n distal tubule (DT) but not d25n DT (dashed outlines). *CDKN1A* and *SOX9* appear to be elevated in d25h/n DT compared to d25n (solid outlines). **C)** Module scoring of 20 canonical PT markers identified PT-marker-expressing cells in the injured d25h/n DT, consistent with mis-clustering of injured PT cells. **D)** An independent 'validation' PT marker set supports the presence of PT-marker-expressing cells in the injured d25h/n DT. **E)** Expression of the validation PT marker set is reduced in d25h/n DT after reclassification. **F)** UMAP showing the original cell labels for the redefined PT cluster **G)** Expression of the custom injury module gene set in d25n and d25h/n PT clusters with exploratory statistical analysis using Wilcoxon rank sum tests showing a significant increase in *ICAM1, PROM1, SOX9 and SPP1*; note that *SOX9* and *PROM1* did not reach significance in pseudobulk analysis of replicate means. *A second, more conservative PT reclassification analysis was also performed using a reference-anchored UCell-based approach described in Supplementary Methods above; results and full code are available in the GitHub repository linked below.

https://github.com/MonashBioinformaticsPlatform/sc-hyp-org.

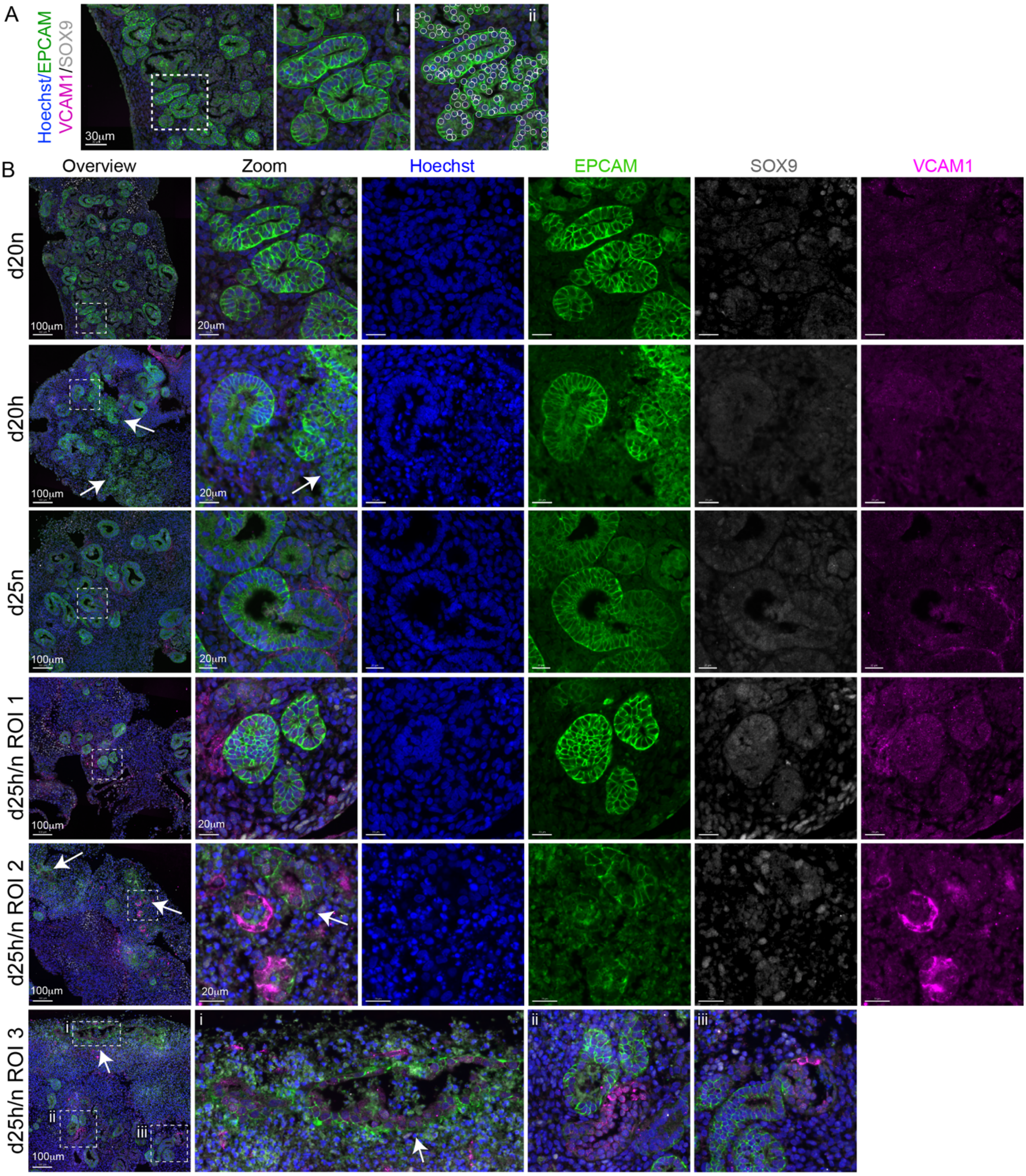

Supplementary Figure 6. Additional VCAM1 and SOX9 staining and segmentation workflow. **A**) Overview of the image analysis workflow used to define nephron structures and associated nuclei. Tubular regions were segmented using LTL or EPCAM staining. While EPCAM is enriched in distal tubules, proximal tubules express sufficient levels to enable robust segmentation. **B**) Representative images of SOX9 (grey) and VCAM1 (magenta) staining across organoid conditions; nuclei (blue), tubules (EPCAM, green). Overview images (left) provide context for magnified views (dashed outline). SOX9 expression is generally low within tubules, with higher levels observed in some stromal regions. VCAM1 expression is low in tubular structures in d20n, d25n, and d20h organoids, but increases in d25h/n organoids. Most tubules show low but elevated VCAM1 expression (e.g. d25h/n ROI 1), while a subset of tubules exhibit markedly higher VCAM1 levels (e.g. d25h/n ROI 2, ROI 3 ii; glomerulus in ROI 3 iii). EPCAM highlights areas of epithelial disruption in d20h (arrows), characterised by diffuse and disorganised signal, with similar regions persisting in d25h/n (arrows).

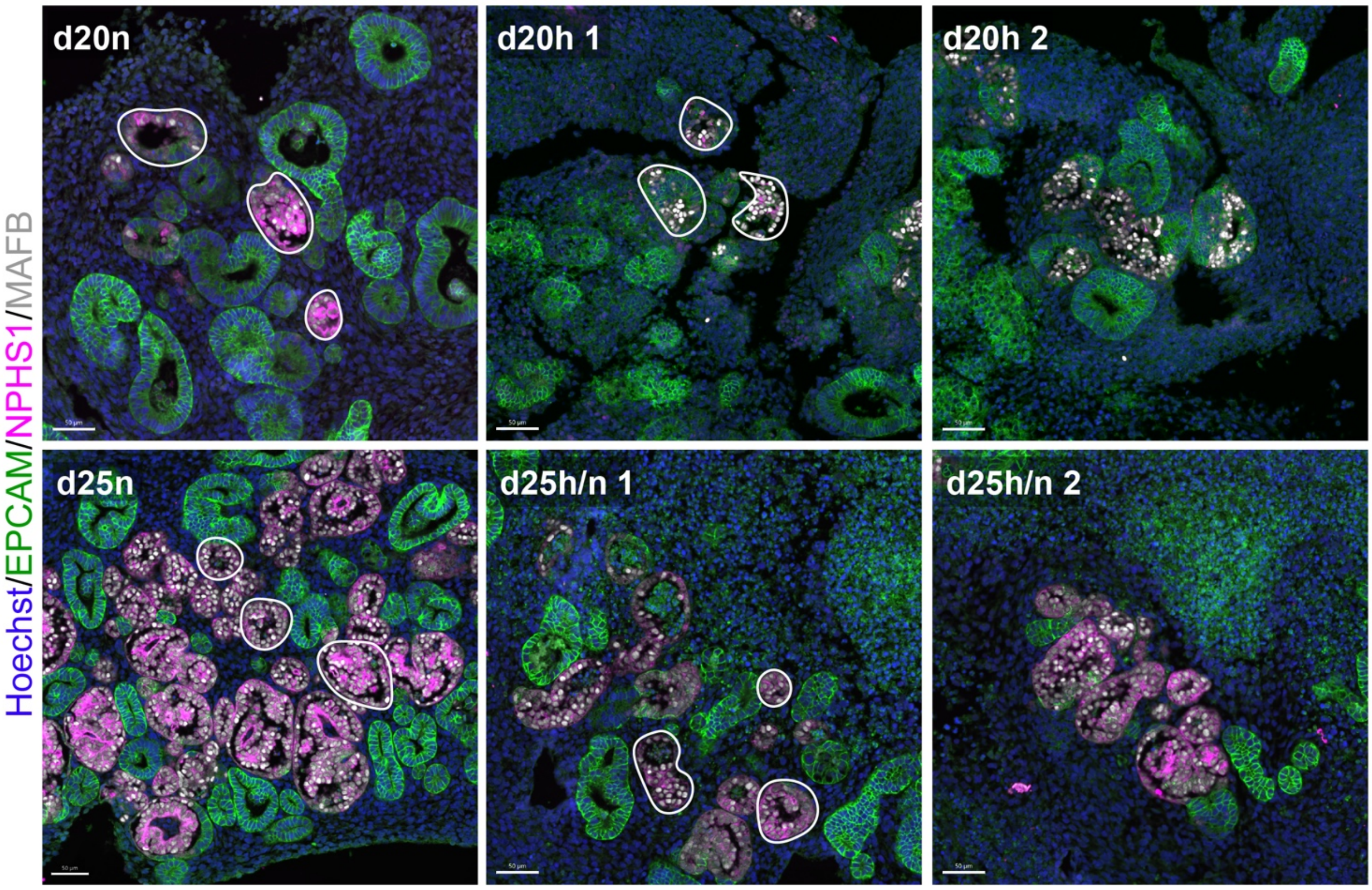

Supplementary Figure 7. Podocyte marker expression and structural changes following hypoxic injury. Representative images of NPHS1 (magenta) and MAFB (grey) immunostaining across conditions (d20n, d20h, d25n, d25h/n), with EPCAM (green) marking epithelial/tubular structures. White outlines indicate example segmentations of $NPHS1^{+}/MAFB^{+}$ structures used for quantification. In control organoids, NPHS1 expression is readily detected within $MAFB^{+}$ podocyte structures at both d20n and d25n, with well-defined glomerular and tubular architecture. Following hypoxic injury (d20h), NPHS1 signal is reduced within $MAFB^{+}$ structures, despite retention of MAFB expression, consistent with early podocyte injury. NPHS1 expression levels recover in d25h/n organoids. EPCAM staining highlights disruption of tubular architecture in d20h organoids, with diffuse epithelial signal and poorly defined structures not observed in d20n controls. Residual regions of disrupted epithelium persist in d25h/n organoids, whereas d25n controls display densely packed, morphologically intact tubules and glomerular structures with minimal intervening stroma. Scale bars, 50 µm.

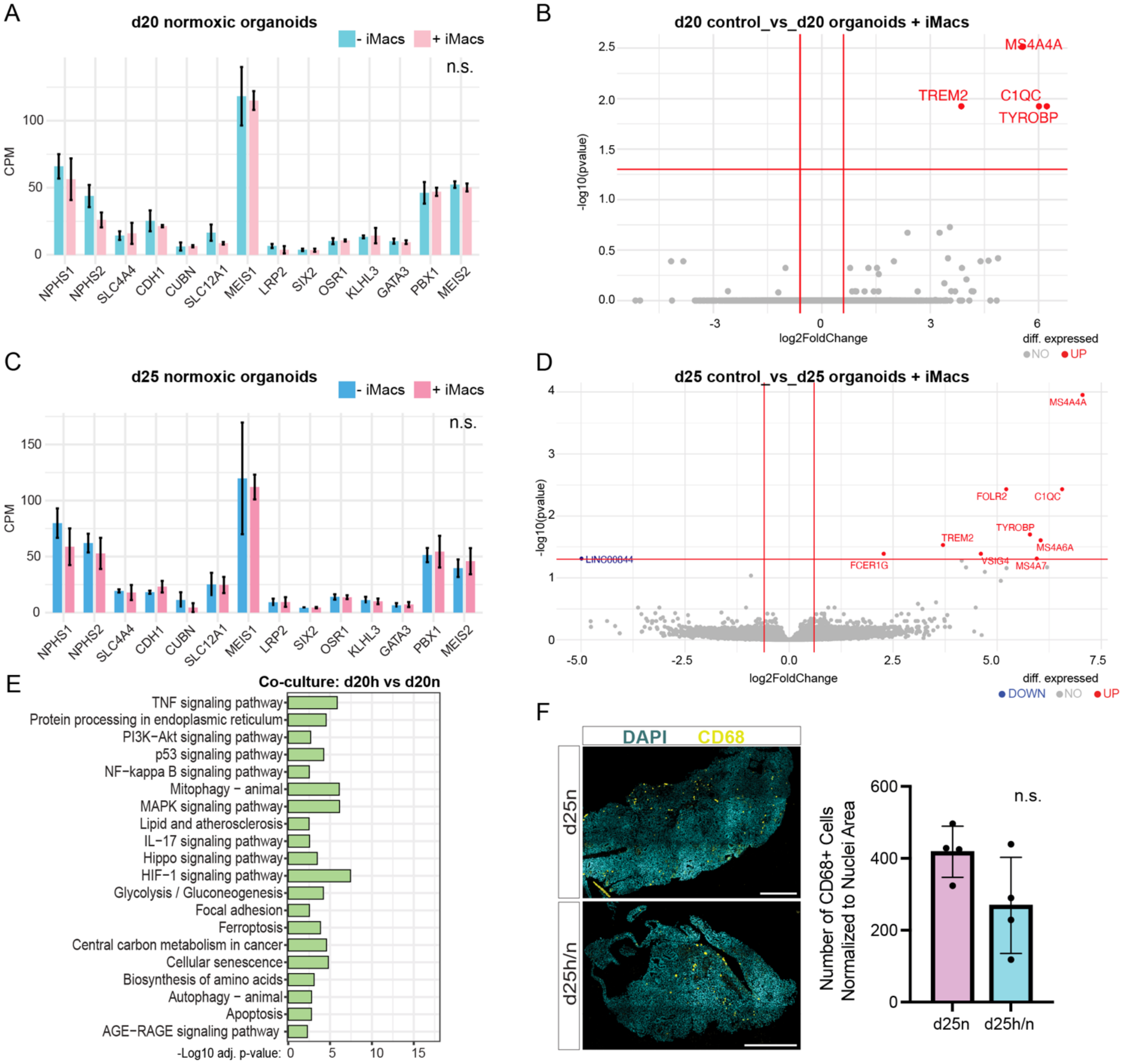

Supplementary Figure 8. Bulk RNAseq analysis of kidney organoids containing iMacs. A) Expression levels of cell type markers in d20 normoxic organoids with- and without-macrophages (+/-iMacs). Error bars represent standard deviation of 3 biological replicates. Two-tailed t-test was performed, n = 3; ns. non-significant. **B)** Volcano plot of DE genes (LogFC>1, adj. p<0.05) between d20 normoxic organoids +/-iMacs. Red indicates upregulated genes in +iMac group; non-significant changes in grey. **C)** Expression levels of cell type markers in d25 normoxic organoids +/-iMacs (n=3). **D)** DE genes between d25 normoxic organoids with and without macrophages (LogFC>1, adj. p<0.05). **E)** Upregulated pathways in d20 iMac-organoids after hypoxia. DE genes (p < 0.05) were used to perform enrichment analysis with DAVID; pathways with FDR<0.05 displayed. **F)** Representative images of d25 hypoxic and normoxic organoids +iMacs labelled with CD68. Scale bar = 500 µm. Quantification of CD68 positive cells normalized to DAPI, n=4, ns. non-significant.

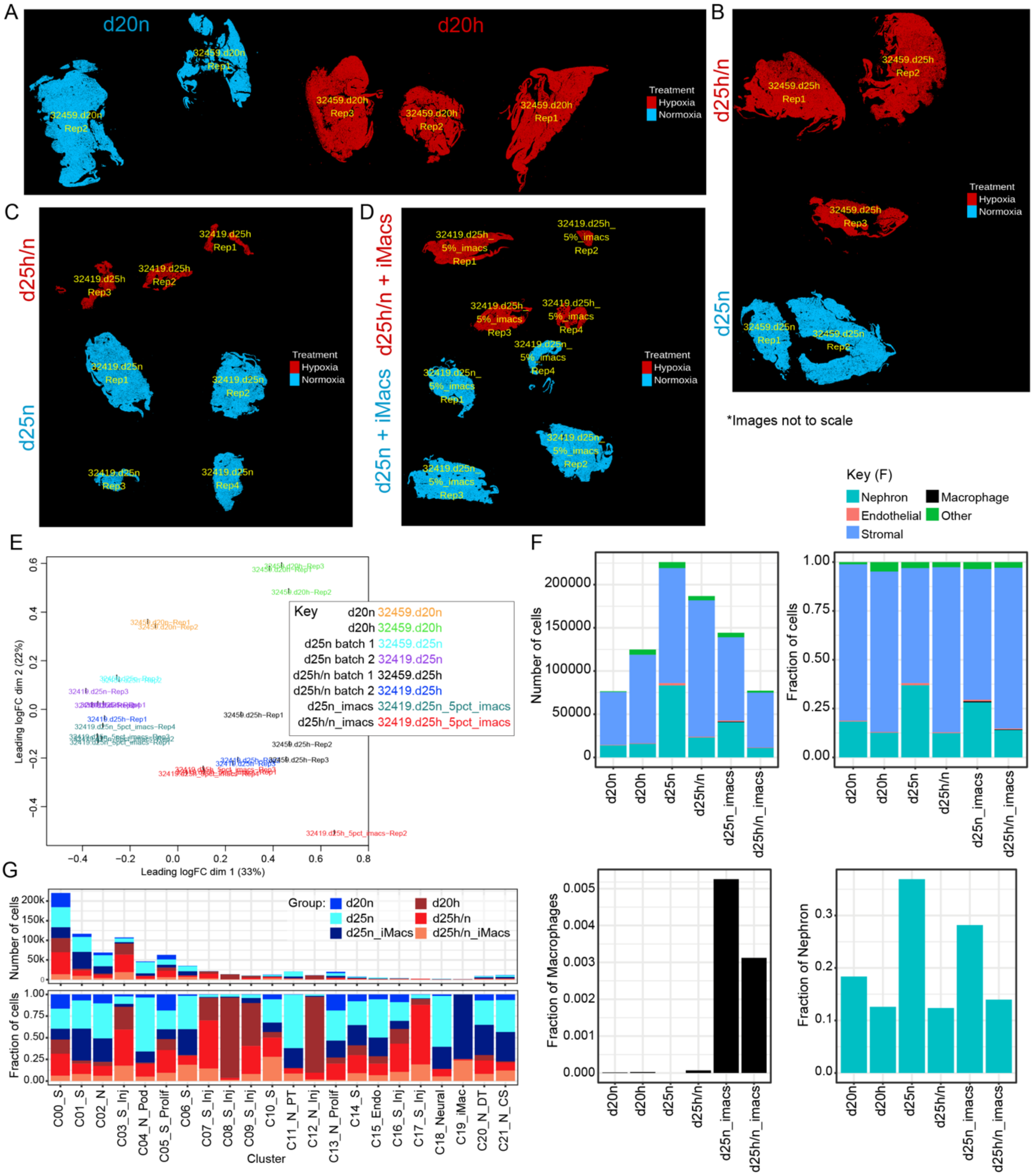

Supplementary Figure 9. Xenium spatial analysis of kidney organoids co-cultured with iMacs. A-D) Images of tissue outlines and experimental groups for all spatial data. Stage and experimental groups indicated as per labels, images not to scale. **E**) MDS plot showing all samples by batch and group. One d25h/n sample (32419.d25h−Rep1) clustered with d25n controls and was excluded from differential expression analysis. **F**) Number and fraction of cells in each major cell type or lineage by sample group. **G**)Absolute number of cells and fractional contributions to each cluster from each sample group according to colours and labels in the colour key.

Supplementary Table 1. Cell Proportions: hypoxia_d20 vs control_d20 (t-test)

BaselineProp.clusters	BaselineProp.Freq	PropMean D20h	PropMean D20n	P.Value	FDR
13_Endothelial	0.0252	0.0038	0.0649	0.0000	0.0000
11_Cellular_Stress	0.0423	0.0531	0.0229	0.0001	0.0005
0_Stroma	0.0947	0.1154	0.0566	0.0003	0.0012
8_Podocyte	0.0617	0.0740	0.0405	0.0032	0.0114
5_Distal_Tubule	0.1234	0.1420	0.0887	0.0082	0.0205
4_Stroma	0.0973	0.1121	0.0695	0.0096	0.0205
6_Proliferating	0.0695	0.0571	0.0930	0.0103	0.0205
1_Stroma	0.1031	0.0899	0.1269	0.0431	0.0737
3_Stroma	0.0864	0.0751	0.1071	0.0474	0.0737
2_Stroma	0.1123	0.0987	0.1365	0.0635	0.0889
9_Neural	0.0423	0.0379	0.0504	0.1409	0.1794
12_Proximal_Tubule	0.0229	0.0267	0.0165	0.2808	0.3276
7_Myocyte	0.0595	0.0567	0.0637	0.4149	0.4469
10_Early_Nephron	0.0593	0.0574	0.0627	0.6153	0.6153

Supplementary Table 2. Cell Proportions: injured_d25 vs control_d25 (t-test)

BaselineProp.clusters	BaselineProp.Freq	PropMean D25h/n	PropMean D25n/n	P.Value	FDR
10_Early_Nephron	0.0289	0.0187	0.0547	0.0001	0.0008
0_Stroma	0.1549	0.1827	0.0830	0.0004	0.0025
4_Stroma	0.0889	0.0675	0.1429	0.0008	0.0038
9_Neural	0.0539	0.0400	0.0883	0.0026	0.0091
11_Cellular_Stress	0.0389	0.0459	0.0195	0.0033	0.0092
13_Endothelial	0.0134	0.0089	0.0239	0.0052	0.0122
5_Distal_Tubule	0.0598	0.0495	0.0851	0.0154	0.0309
6_Proliferating	0.0949	0.1059	0.0668	0.0198	0.0324
12_Proximal_Tubule	0.0136	0.0098	0.0236	0.0208	0.0324
1_Stroma	0.1317	0.1471	0.0946	0.0289	0.0405
8_Podocyte	0.0337	0.0360	0.0288	0.2566	0.3209
7_Myocyte	0.0839	0.0883	0.0726	0.2751	0.3209
2_Stroma	0.0990	0.0958	0.1091	0.4671	0.5030
3_Stroma	0.1044	0.1039	0.1073	0.9145	0.9145

Supplementary Table 3. Acute and persistent markers across organoid and clinical AKI

Marker Gene	Description	Species	Reference
**Ischemia Reperfusion Injury**			
**GDF15**	Serum levels correlate with AKI severity<br>↑ in unilateral IRI and UUO; ↑ in HK-2 cells after oxidative stress and hypoxia	Human<br>Mouse & human cell	(43)<br>(44)
**CXCL1**	↑ in plasma of AKI patients	Human	(45)
**HMOX1**	↑ in urine & plasma of AKI patients	Human	(46, 47)
**FOS**	Early induced gene in renal ischemia	Mouse	(48)
**JUN**	↑ DNA binding in post-ischemic kidneys	Rat	(49)
**CDKN1A**	Expression ↑ in kidney after AKI	Mouse/rat	(50)
**BNIP3**	↑ in PT after AKI	Rat	(51)
**CCN1**	↑ in urine after renal ischemia	Mouse/Rat	(52)
**Persistent injury associated markers/AKI-CKD transition**			
**CXCL1**	↑ in urine or kidney during IgA nephropathy and diabetic nephropathy	Human	(53, 54)
**C3**	↑ in plasma of CKD patients	Human	(55)
**ICAM1**	Serum levels correlate with stage of CKD	Human	(56)
**STAT3**	pSTAT3 ↑ in CKD tubules & interstitium	Human	(57)
**SAA1**	Serum levels increase in CKD	Human	(58)
**CCL2**	Urinary marker of subclinical AKI linked to increased risk of CKD, ↑ urinary CCL2:creatinine linked to renal decline	Human	(59, 60)
**LCN2/NGAL**	Prognostic marker for CKD progression,<br>↑ urinary NGAL correlates with ↓ eGFR	Human	(61)
**IL32**	Correlates with severity of chronic obstructive pulmonary disease, ↑ in PT of rejecting kidney allograft biopsy	Human	(62, 63)
**COL4A4**	↑ urinary excretion associated with ↓ renal function in diabetic kidney disease	Human	(64)
**VCAM1**	Serum levels correlate with stage of CKD<br>PT expression drives AKI-CKD transition	Human<br>Mouse	(56)<br>(65)
**FN1**	↑ in urine of patients with diabetic nephropathy and glomerular diseases<br>Upregulated in CKD	Human<br>Mouse	(66)<br>(67)
**MMP7**	↑ mRNA in kidney tissue of CKD patients	Human	(68)
**MMP9**	↑ urine levels in stage 3-4 CKD patients	Human	(69)
**GDF15**	Increased plasma correlated with increased risk of CKD progression	Human	(70)
**CCN2/CTGF**	N-term. fragment ↑ in urine during CKD<br>CCN2 vaccine attenuates renal fibrosis	Human<br>Mouse	(71)<br>(72)
**KRT8**	↑ in injured tubules<br>↑ in urine of UUO models	Human<br>Mouse	(73)<br>(73)
**KRT18**	↑ in urine of AKI and stage 5 CKD patients; ↑ in serum stages 3-5 CKD	Human	(73)
**SPINK1**	No clear link to CKD; Associated with chronic inflammation and cancer.	Human	(74)
**SPP1**	↑ urine linked to ↓ kidney function in CKD	Human	(75)
**SOD2**	Protein ↓ stage 1-4 CKD, ↑ stage 5 CKD	Human	(76)
**HSPA5**	↑ in CKD and in UUO model	Human	(77)
**CXCL2**	Linked to PT maladaptive repair after IRI	Mouse	(78)
**S100A8/9**	Linked to PT maladaptive repair after IRI	Mouse	(78)
**CCN1**	↑ in tubules in UUO, blockade attenuates kidney fibrosis	Mouse	(79)
**HIPK2**	Promotes kidney fibrosis	Mouse	(80)

Abbreviations: IRI ischemia-reperfusion injury, UUO unilateral ureter obstruction model, ↑ increased, ↓ decreased, PT proximal tubule, eGFR estimated glomerular filtration rate.

Supplementary References

1. Howden SE, Little MH. Generating Kidney Organoids from Human Pluripotent Stem Cells Using Defined Conditions. Methods Mol Biol. 2020;2155:183-92.
2. Takasato M, Er PX, Becroft M, Vanslambrouck JM, Stanley EG, Elefanty AG, et al. Directing human embryonic stem cell differentiation towards a renal lineage generates a self-organizing kidney. Nature Cell Biology. 2014;16(1):118-26.
3. Takasato M, Er PX, Chiu HS, Maier B, Baillie GJ, Ferguson C, et al. Kidney organoids from human iPS cells contain multiple lineages and model human nephrogenesis. Nature. 2015;526(7574):564-8.
4. Joshi K, Elso C, Motazedian A, Labonne T, Schiesser JV, Cameron F, et al. Induced pluripotent stem cell macrophages present antigen to proinsulin-specific T cell receptors from donor-matched islet-infiltrating T cells in type 1 diabetes. Diabetologia. 2019;62(12):2245-51.
5. Ng ES, Davis RP, Hatzistavrou T, Stanley EG, Elefanty AG. Directed differentiation of human embryonic stem cells as spin embryoid bodies and a description of the hematopoietic blast colony forming assay. Curr Protoc Stem Cell Biol. 2008;Chapter 1:Unit 1D 3.
6. Rajab N, Angel PW, Deng Y, Gu J, Jameson V, Kurowska-Stolarska M, et al. An integrated analysis of human myeloid cells identifies gaps in in vitro models of in vivo biology. Stem Cell Reports. 2021;16(6):1629-43.
7. Yanagimachi MD, Niwa A, Tanaka T, Honda-Ozaki F, Nishimoto S, Murata Y, et al. Robust and highly-efficient differentiation of functional monocytic cells from human pluripotent stem cells under serum- and feeder cell-free conditions. PLoS One. 2013;8(4):e59243.
8. Dobin A, Davis CA, Schlesinger F, Drenkow J, Zaleski C, Jha S, et al. STAR: ultrafast universal RNA-seq aligner. Bioinformatics. 2013;29(1):15-21.
9. Li H, Handsaker B, Wysoker A, Fennell T, Ruan J, Homer N, et al. The Sequence Alignment/Map format and SAMtools. Bioinformatics. 2009;25(16):2078-9.
10. Smith T, Heger A, Sudbery I. UMI-tools: modeling sequencing errors in Unique Molecular Identifiers to improve quantification accuracy. Genome Res. 2017;27(3):491-9.
11. Liao Y, Smyth GK, Shi W. featureCounts: an efficient general purpose program for assigning sequence reads to genomic features. Bioinformatics. 2014;30(7):923-30.
12. Durinck S, Spellman PT, Birney E, Huber W. Mapping identifiers for the integration of genomic datasets with the R/Bioconductor package biomaRt. Nat Protoc. 2009;4(8):1184-91.
13. Ritchie ME, Phipson B, Wu D, Hu Y, Law CW, Shi W, et al. limma powers differential expression analyses for RNA-sequencing and microarray studies. Nucleic acids research. 2015;43(7):e47-e.
14. Law CW, Chen Y, Shi W, Smyth GK. voom: Precision weights unlock linear model analysis tools for RNA-seq read counts. Genome biology. 2014;15(2):1-17.
15. Powell D. Degust: interactive RNA-seq analysis. Drpowell/Degust. 2015;4(1):4.1.
16. Sherman BT, Hao M, Qiu J, Jiao X, Baseler MW, Lane HC, et al. DAVID: a web server for functional enrichment analysis and functional annotation of gene lists (2021 update). Nucleic acids research. 2022;50(W1):W216-W21.
17. Subramanian A, Tamayo P, Mootha VK, Mukherjee S, Ebert BL, Gillette MA, et al. Gene set enrichment analysis: a knowledge-based approach for interpreting genome-wide expression profiles. Proceedings of the National Academy of Sciences. 2005;102(43):15545-50.
18. Liberzon A, Subramanian A, Pinchback R, Thorvaldsdottir H, Tamayo P, Mesirov JP. Molecular signatures database (MSigDB) 3.0. Bioinformatics. 2011;27(12):1739-40.
19. Zheng GX, Terry JM, Belgrader P, Ryvkin P, Bent ZW, Wilson R, et al. Massively parallel digital transcriptional profiling of single cells. Nat Commun. 2017;8:14049.
20. Hao Y, Hao S, Andersen-Nissen E, Mauck WM, 3rd, Zheng S, Butler A, et al. Integrated analysis of multimodal single-cell data. Cell. 2021;184(13):3573-87 e29.

21. Hafemeister C, Satija R. Normalization and variance stabilization of single-cell RNA-seq data using regularized negative binomial regression. Genome Biol. 2019;20(1):296.
22. Zappia L, Oshlack A. Clustering trees: a visualization for evaluating clusterings at multiple resolutions. Gigascience. 2018;7(7).
23. Xie Z, Bailey A, Kuleshov MV, Clarke DJB, Evangelista JE, Jenkins SL, et al. Gene Set Knowledge Discovery with Enrichr. Curr Protoc. 2021;1(3):e90.
24. Wilson SB, Howden SE, Vanslambrouck JM, Dorison A, Alquicira-Hernandez J, Powell JE, et al. DevKidCC allows for robust classification and direct comparisons of kidney organoid datasets. Genome Med. 2022;14(1):19.
25. Phipson B, Sim CB, Porrello ER, Hewitt AW, Powell J, Oshlack A. propeller: testing for differences in cell type proportions in single cell data. Bioinformatics. 2022;38(20):4720-6.
26. Squair JW, Gautier M, Kathe C, Anderson MA, James ND, Hutson TH, et al. Confronting false discoveries in single-cell differential expression. Nat Commun. 2021;12(1):5692.
27. Ritchie ME, Phipson B, Wu D, Hu Y, Law CW, Shi W, et al. limma powers differential expression analyses for RNA-sequencing and microarray studies. Nucleic Acids Res. 2015;43(7):e47.
28. Robinson MD, McCarthy DJ, Smyth GK. edgeR: a Bioconductor package for differential expression analysis of digital gene expression data. Bioinformatics. 2010;26(1):139-40.
29. Humphreys BD. Mechanisms of Renal Fibrosis. Annu Rev Physiol. 2018;80:309-26.
30. Teo GC, Polasky DA, Yu F, Nesvizhskii AI. Fast Deisotoping Algorithm and Its Implementation in the MSFragger Search Engine. J Proteome Res. 2021;20(1):498-505.
31. Kong AT, Leprevost FV, Avtonomov DM, Mellacheruvu D, Nesvizhskii AI. MSFragger: ultrafast and comprehensive peptide identification in mass spectrometry–based proteomics. Nature Methods. 2017;14(5):513-20.
32. Yu F, Haynes SE, Nesvizhskii AI. IonQuant Enables Accurate and Sensitive Label-Free Quantification With FDR-Controlled Match-Between-Runs. Mol Cell Proteomics. 2021;20:100077.
33. Shah AD, Goode RJA, Huang C, Powell DR, Schittenhelm RB. LFQ-Analyst: An Easy-To-Use Interactive Web Platform To Analyze and Visualize Label-Free Proteomics Data Preprocessed with MaxQuant. Journal of Proteome Research. 2020;19(1):204-11.
34. Creek DJ, Jankevics A, Breitling R, Watson DG, Barrett MP, Burgess KEV. Toward Global Metabolomics Analysis with Hydrophilic Interaction Liquid Chromatography–Mass Spectrometry: Improved Metabolite Identification by Retention Time Prediction. Analytical Chemistry. 2011;83(22):8703-10.
35. Chambers MC, Maclean B, Burke R, Amodei D, Ruderman DL, Neumann S, et al. A cross-platform toolkit for mass spectrometry and proteomics. Nature Biotechnology. 2012;30(10):918-20.
36. Creek DJ, Jankevics A, Burgess KE, Breitling R, Barrett MP. IDEOM: an Excel interface for analysis of LC-MS-based metabolomics data. Bioinformatics. 2012;28(7):1048-9.
37. Scheltema RA, Jankevics A, Jansen RC, Swertz MA, Breitling R. PeakML/mzMatch: a file format, Java library, R library, and tool-chain for mass spectrometry data analysis. Anal Chem. 2011;83(7):2786-93.
38. Wilhelm D, Washburn LL, Truong V, Fellous M, Eicher EM, Koopman P. Antagonism of the testis- and ovary-determining pathways during ovotestis development in mice. Mech Dev. 2009;126(5-6):324-36.
39. Masaki T, Foti R, Hill PA, Ikezumi Y, Atkins RC, Nikolic-Paterson DJ. Activation of the ERK pathway precedes tubular proliferation in the obstructed rat kidney. Kidney Int. 2003;63(4):1256-64.
40. Cable DM, Murray E, Zou LS, Goeva A, Macosko EZ, Chen F, et al. Robust decomposition of cell type mixtures in spatial transcriptomics. Nat Biotechnol. 2022;40(4):517-26.

41. Law CW, Chen Y, Shi W, Smyth GK. voom: Precision weights unlock linear model analysis tools for RNA-seq read counts. Genome Biol. 2014;15(2):R29.
42. Smyth GK. Linear models and empirical bayes methods for assessing differential expression in microarray experiments. Stat Appl Genet Mol Biol. 2004;3:Article3.
43. Lim JH, Jeon Y, Ahn JS, Kim S, Kim DK, Lee JP, et al. GDF-15 Predicts In-Hospital Mortality of Critically Ill Patients with Acute Kidney Injury Requiring Continuous Renal Replacement Therapy: A Multicenter Prospective Study. J Clin Med. 2021;10(16).
44. Sawasawa T, Lin JD, Wang YH, Chen KJ, Yang YM, Hu SW, et al. Elevated serum GDF15 level as an early indicator of proximal tubular cell injury in acute kidney injury. Life Sci. 2024;357:123093.
45. Li X, Zhou X, Ping X, Zhao X, Kang H, Zhang Y, et al. Combined Plasma Olink Proteomics and Transcriptomics Identifies CXCL1 and TNFRSF12A as Potential Predictive and Diagnostic Inflammatory Markers for Acute Kidney Injury. Inflammation. 2024;47(5):1547-63.
46. Zager RA, Johnson AC, Becker K. Plasma and urinary heme oxygenase-1 in AKI. J Am Soc Nephrol. 2012;23(6):1048-57.
47. Billings FTt, Yu C, Byrne JG, Petracek MR, Pretorius M. Heme Oxygenase-1 and Acute Kidney Injury following Cardiac Surgery. Cardiorenal Med. 2014;4(1):12-21.
48. Ouellette AJ, Malt RA, Sukhatme VP, Bonventre JV. Expression of two "immediate early" genes, Egr-1 and c-fos, in response to renal ischemia and during compensatory renal hypertrophy in mice. J Clin Invest. 1990;85(3):766-71.
49. Morooka H, Bonventre JV, Pombo CM, Kyriakis JM, Force T. Ischemia and Reperfusion Enhance ATF-2 and c-Jun Binding to cAMP Response Elements and to an AP-1 Binding Site from the c-jun Promoter (∗). Journal of Biological Chemistry. 1995;270(50):30084-92.
50. Megyesi J, Udvarhelyi N, Safirstein RL, Price PM. The p53-independent activation of transcription of p21 WAF1/CIP1/SDI1 after acute renal failure. Am J Physiol. 1996;271(6 Pt 2):F1211-6.
51. Ishihara M, Urushido M, Hamada K, Matsumoto T, Shimamura Y, Ogata K, et al. Sestrin-2 and BNIP3 regulate autophagy and mitophagy in renal tubular cells in acute kidney injury. Am J Physiol Renal Physiol. 2013;305(4):F495-509.
52. Muramatsu Y, Tsujie M, Kohda Y, Pham B, Perantoni AO, Zhao H, et al. Early detection of cysteine rich protein 61 (CYR61, CCN1) in urine following renal ischemic reperfusion injury. Kidney Int. 2002;62(5):1601-10.
53. Zhao Y, Zhu L, Zhou T, Zhang Q, Shi S, Liu L, et al. Urinary CXCL1: a novel predictor of IgA nephropathy progression. PLoS One. 2015;10(3):e0119033.
54. Tang H, Yang M, Liu Y, Liu H, Sun L, Song P. The CXCL1-CXCR2 Axis Mediates Tubular Injury in Diabetic Nephropathy Through the Regulation of the Inflammatory Response. Front Physiol. 2021;12:782677.
55. Bao X, Borné Y, Muhammad IF, Schulz CA, Persson M, Orho-Melander M, et al. Complement C3 and incident hospitalization due to chronic kidney disease: a population-based cohort study. BMC Nephrol. 2019;20(1):61.
56. Attalah MF, Adel LA, Fahmy DE. Diagnostic and prognostic values of adhesion molecules VCAM-1, ICAM-1 and C-reactive protein in Egyptian patients with chronic kidney disease. Egypt J Immunol. 2011;18(2):59-66.
57. Ajay AK, Zhao L, Vig S, Fujiwara M, Thakurela S, Jadhav S, et al. Deletion of STAT3 from Foxd1 cell population protects mice from kidney fibrosis by inhibiting pericytes trans-differentiation and migration. Cell Reports. 2022;38(10).

58. Zhang F, Zhou X, Zou H, Liu L, Li X, Ruan Y, et al. SAA1 is transcriptionally activated by STAT3 and accelerates renal interstitial fibrosis by inducing endoplasmic reticulum stress. Experimental Cell Research. 2021;408(1):112856.
59. Zhang WR, Craven TE, Malhotra R, Cheung AK, Chonchol M, Drawz P, et al. Kidney Damage Biomarkers and Incident Chronic Kidney Disease During Blood Pressure Reduction: A Case-Control Study. Ann Intern Med. 2018;169(9):610-8.
60. Nadkarni GN, Rao V, Ismail-Beigi F, Fonseca VA, Shah SV, Simonson MS, et al. Association of Urinary Biomarkers of Inflammation, Injury, and Fibrosis with Renal Function Decline: The ACCORD Trial. Clin J Am Soc Nephrol. 2016;11(8):1343-52.
61. Tomonaga Y, Szucs T, Ambühl P, Nock S, Risch M, Risch L. Insights on urinary NGAL obtained in a primary care setting. Clinica Chimica Acta. 2012;413(7):733-9.
62. Rong B, Fu T, Rong C, Liu W, Li K, Liu H. Correlation between serum IL-32 concentration and clinical parameters of stable COPD: a retrospective clinical analysis. Scientific Reports. 2020;10(1):12092.
63. Wu H, Malone AF, Donnelly EL, Kirita Y, Uchimura K, Ramakrishnan SM, et al. Single-Cell Transcriptomics of a Human Kidney Allograft Biopsy Specimen Defines a Diverse Inflammatory Response. J Am Soc Nephrol. 2018;29(8):2069-80.
64. Araki S, Haneda M, Koya D, Isshiki K, Kume S, Sugimoto T, et al. Association between urinary type IV collagen level and deterioration of renal function in type 2 diabetic patients without overt proteinuria. Diabetes Care. 2010;33(8):1805-10.
65. Melchinger I, Guo K, Li X, Guo J, Cantley LG, Xu L. VCAM-1 mediates proximal tubule-immune cell cross talk in failed tubule recovery during AKI-to-CKD transition. Am J Physiol Renal Physiol. 2024;327(4):F610-f22.
66. Ren L, Blanchette JB, White LR, Clark SA, Heffner DJ, Tibbles LA, et al. Soluble fibronectin induces chemokine gene expression in renal tubular epithelial cells. Kidney International. 2005;68(5):2111-20.
67. Huang R, Fu P, Ma L. Kidney fibrosis: from mechanisms to therapeutic medicines. Signal Transduction and Targeted Therapy. 2023;8(1):129.
68. Zheng CM, Lu KC, Chen YJ, Li CY, Lee YH, Chiu HW. Matrix metalloproteinase-7 promotes chronic kidney disease progression via the induction of inflammasomes and the suppression of autophagy. Biomed Pharmacother. 2022;154:113565.
69. Pawlak K, Mysliwiec M, Pawlak D. Peripheral blood level alterations of MMP-2 and MMP-9 in patients with chronic kidney disease on conservative treatment and on hemodialysis. Clinical Biochemistry. 2011;44(10):838-43.
70. Nair V, Robinson-Cohen C, Smith MR, Bellovich KA, Bhat ZY, Bobadilla M, et al. Growth Differentiation Factor-15 and Risk of CKD Progression. J Am Soc Nephrol. 2017;28(7):2233-40.
71. Nguyen TQ, Gerritsen KG, Goldschmeding R. Does High or Low Urinary Connective Tissue Growth Factor Predict CKD? American Journal of Kidney Diseases. 2011;58(4):680-1.
72. Nakayama T, Azegami T, Hayashi K, Hishikawa A, Yoshimoto N, Nakamichi R, et al. Vaccination against connective tissue growth factor attenuates the development of renal fibrosis. Scientific Reports. 2022;12(1):10933.
73. Djudjaj S, Papasotiriou M, Bülow RD, Wagnerova A, Lindenmeyer MT, Cohen CD, et al. Keratins are novel markers of renal epithelial cell injury. Kidney International. 2016;89(4):792-808.
74. Timofte AD, Caruntu I-D, Covic AC, Hancianu M, Girlescu N, Chifu MB, et al. Renal Function Parameters in Distinctive Molecular Subtypes of Prostate Cancer. Cancers [Internet]. 2023; 15(20).
75. Steinbrenner I, Sekula P, Kotsis F, von Cube M, Cheng Y, Nadal J, et al. Association of osteopontin with kidney function and kidney failure in chronic kidney disease patients: the GCKD study. Nephrol Dial Transplant. 2023;38(6):1430-8.

76. Krueger K, Shen J, Maier A, Tepel M, Scholze A. Lower Superoxide Dismutase 2 (SOD2) Protein Content in Mononuclear Cells Is Associated with Better Survival in Patients with Hemodialysis Therapy. Oxid Med Cell Longev. 2016;2016:7423249.
77. Bhreathnach U, Griffin B, Brennan E, Ewart L, Higgins D, Murphy M. Profibrotic IHG-1 complexes with renal disease associated HSPA5 and TRAP1 in mitochondria. Biochimica et Biophysica Acta (BBA) - Molecular Basis of Disease. 2017;1863(4):896-906.
78. Balzer MS, Doke T, Yang YW, Aldridge DL, Hu H, Mai H, et al. Single-cell analysis highlights differences in druggable pathways underlying adaptive or fibrotic kidney regeneration. Nat Commun. 2022;13(1):4018.
79. Lai CF, Chen YM, Chiang WC, Lin SL, Kuo ML, Tsai TJ. Cysteine-rich protein 61 plays a proinflammatory role in obstructive kidney fibrosis. PLoS One. 2013;8(2):e56481.
80. Jin Y, Ratnam K, Chuang PY, Fan Y, Zhong Y, Dai Y, et al. A systems approach identifies HIPK2 as a key regulator of kidney fibrosis. Nat Med. 2012;18(4):580-8.
